# Monitoring Membrane Protein Folding Assisted by Insertases and Translocases Using AFM-Based Single-Molecule Force Spectroscopy

**DOI:** 10.1021/acs.chemrev.5c00960

**Published:** 2026-05-27

**Authors:** Tetiana Serdiuk, Johannes Thoma, Daniel J. Müller

**Affiliations:** † Institute of Molecular Systems Biology, 27219 ETH Zürich, Otto-Stern-Weg 3, 8093 Zürich, Switzerland; ‡ Department of Chemistry and Molecular Biology, 3570 University of Gothenburg, Medicinaregatan 7B, 41390 Gothenburg, Sweden; § Department of Biosystems Science and Engineering, ETH Zurich, Klingelbergstrasse 48, 4056 Basel, Switzerland

## Abstract

In this review we discuss how atomic force microscopy (AFM)-based single-molecule force spectroscopy (SMFS) approaches can be applied to monitor the unfolding and folding pathways of individual membrane proteins. Particularly, we focus on the insertion and folding of prokaryotic α-helical and β-barrel membrane proteins and compare their unassisted insertion and folding pathways with those assisted by insertases, translocases, and chaperones. We highlight examples in which SMFS is applied to detect the misfolding of membrane proteins such as induced by the lipid composition of the membrane or resulting from unassisted folding. While SMFS can monitor how soluble and transmembrane chaperones reduce misfolding of structural segments, it can also monitor how insertases and translocases guide their stepwise insertion and folding into membranes until the membrane protein has completed folding. Examples show that the inner membrane insertase YidC inserts structural segments in a random order, whereas the SecYEG translocon inserts transmembrane α-helices sequentially. However, when acting together, SecYEG dominates over YidC, consistent with the role of the translocon in directing membrane protein insertion and folding. Finally, we discuss β-barrel membrane protein folding in the bacterial outer membrane, including the β-barrel assembly machinery (BAM) complex, and how SMFS applied to native outer membrane vesicles provides access to monitor the insertion and folding of membrane proteins in the native-like membrane environment.

## Graphical abstract

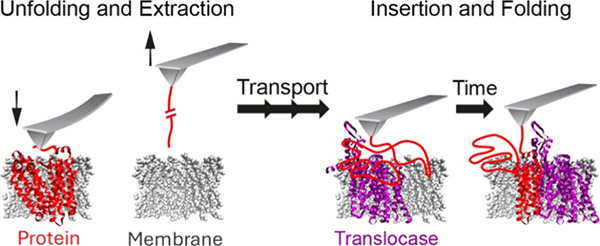

## 1 Introduction

Membrane proteins account for a substantial fraction of the bacterial proteome and are essential for transport, energy conversion, signal transduction, and cell-envelope biogenesis. Thereby, highly conserved molecular machinery governs membrane protein insertion and folding, ensuring accurate targeting and integration into cellular membranes.^1–3^ In Gram-negative bacteria, this process is particularly intricate because the cell envelope comprises an inner membrane, a periplasmic space, and an outer membrane. Following cytosolic synthesis, membrane proteins must be correctly targeted and inserted into their destination membrane to preserve the bilayer integrity. This process further prevents the aggregation of hydrophobic transmembrane segments in the aqueous environment, which is critical for maintaining cellular function and survival. Bacterial membrane protein biogenesis is a multistep pathway that couples protein synthesis to membrane insertion and folding, which is organized by an extensive network of quality-control proteins that includes dedicated targeting factors, chaperones, translocases, and insertases.

From a thermodynamic perspective, membrane protein insertion and folding reflect the balance between the energetic costs of transferring hydrophobic residues into and polar residues across the lipid membrane and the stabilizing interactions formed within the final folded state.^4^ Hydrophobic transmembrane segments of membrane proteins experience a strong driving force to partition into the membrane core, quantitatively described by the apparent free energy of insertion, as measured by biological hydrophobicity scales that reflect sequence-dependent insertion propensities.^5,6^ The framework is grounded in early bulk peptide partitioning experiments, providing a general thermodynamic basis for the underlying membrane association and folding energetics across different topologies.^7,8^ The two-stage model of membrane protein folding, in which transmembrane segments first insert and subsequently associate, extends this framework to the broader principle that membrane protein folding involves the sequential stabilization of hydrophobic segments, followed by cooperative tertiary interactions.^1,8,9^ These thermodynamic principles, extensively reviewed elsewhere,^8–10^ provide the biophysical foundation upon which cellular chaperones shape the energetic landscape and guide membrane protein folding *in vivo* (Figure 1). Although the spontaneous insertion into membranes can be occasionally observed,^11–14^ membrane protein folding *in vivo* is generally facilitated by cellular machineries that lower kinetic barriers, ensure correct topology, and prevent the aggregation of hydrophobic regions in the aqueous cytosolic or periplasmic environments.^15–17^

**Figure 1:**
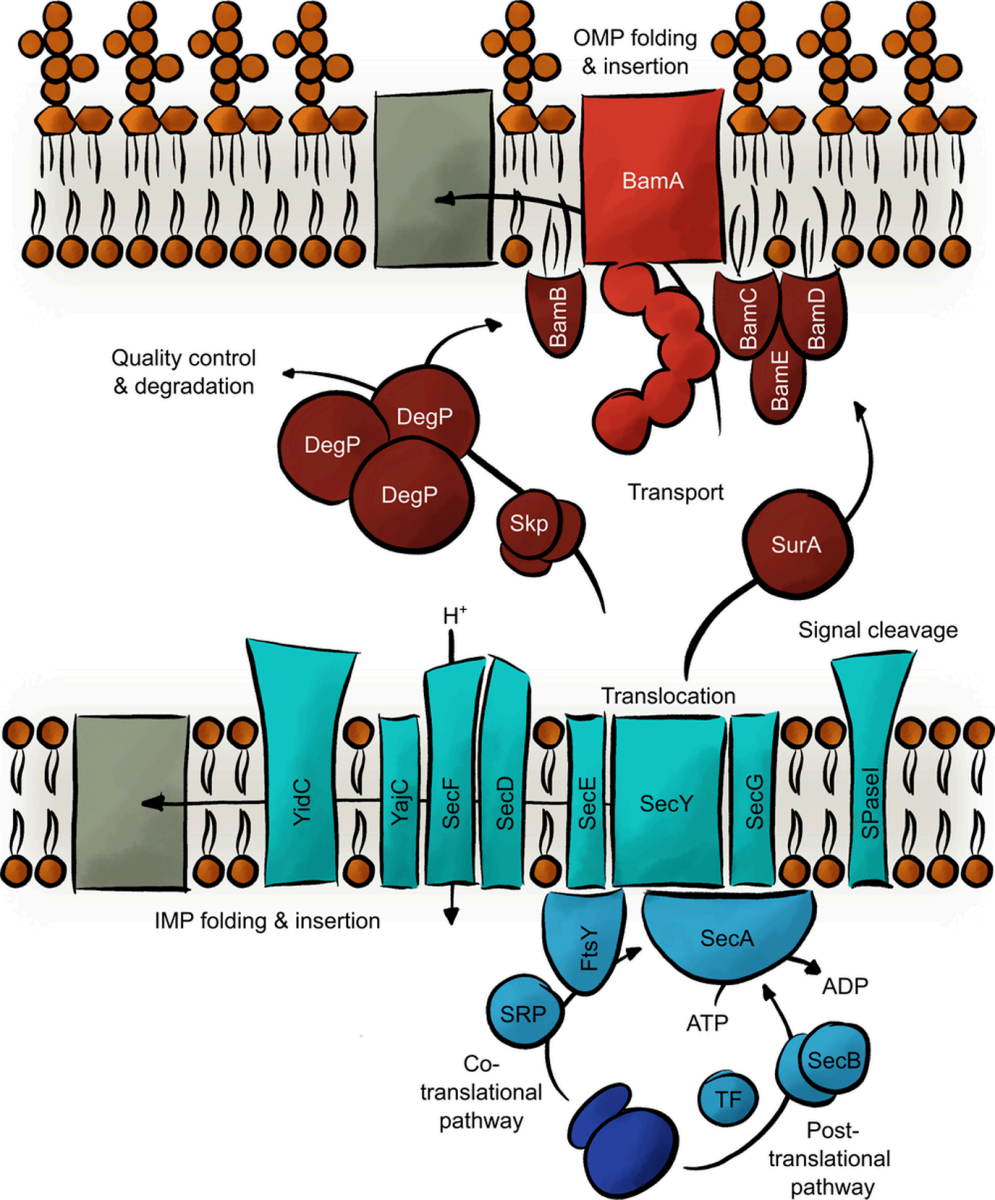
Transport, folding and insertion pathways of inner and outer membrane proteins in Gram-negative bacteria. Nascent polypeptides are sorted at the ribosome into co- or post-translational pathways. In the cotranslational pathway, signal recognition particle (SRP) and FtsY target ribosome–nascent chain complexes to the SecYEG translocon. In the post-translational pathway, cytosolic chaperones (trigger factor (TF), SecB) maintain substrates in an unfolded state for SecA-dependent delivery to SecYEG. SecYEG mediates membrane insertion of inner membrane proteins (IMPs), assisted by YidC and the SecDF–YajC complex, or translocation of outer membrane protein precursors into the periplasm following signal peptide cleavage by SPase I. In the periplasm, SurA transports substrates to the β-barrel assembly machinery (BAM) complex for folding and insertion into the outer membrane, while Skp and DegP contribute to quality control. Adapted with permission from ref.^18^ Copyright 2022 Elsevier.

In cells, the insertion and folding of membrane proteins into lipid membranes is tightly regulated and proceeds through two distinct pathways: cotranslational and post-translational insertion (Figure 1).^19–21^ Pathway selection is initiated at the ribosome during the earliest stages of synthesis. As soon as the nascent polypeptide emerges from the ribosomal exit tunnel, it is immediately received by ribosome-associated factors that determine its targeting. Proteins destined for the cell envelope are distinguished by an amino-terminal signal sequence that emerges first from the ribosomal exit tunnel. The hydrophobicity of this signal peptide is a key determinant of pathway selection, targeting the nascent chain toward either the cotranslational or the post-translational route.^22^ The ribosome-bound chaperone trigger factor (TF) interacts with many emerging polypeptides and prevents premature folding or inappropriate intermolecular interactions.^23^ Substrates containing moderately hydrophobic signal sequences, such as secretory proteins or outer membrane proteins, are directed to the post-translational pathway.^24^ In this route, proteins are fully synthesized in the cytosol before membrane engagement. Following release from the ribosome, soluble holdase chaperones, primarily SecB or TF, bind the unfolded polypeptide.^23,25^ These chaperones maintain substrates in a highly flexible folding-competent state, shielding hydrophobic segments while preventing aggregation or misfolding.^26–28^ Membrane targeting in the post-translational pathway is mediated by the SecB/SecA system. SecB delivers client proteins to SecYEG-associated ATPase SecA, which then drives the ATP-dependent translocation of the unfolded substrate through the SecYEG channel.^16,29,30^

In contrast, for substrates containing sufficiently hydrophobic signal peptides or transmembrane helices, the signal recognition particle (SRP) competes with or displaces TF and binds directly to the exposed hydrophobic segment.^31^ Binding of SRP to the signal peptide induces a transient stalling of translation and promotes formation of a ribosome-nascent chain-SRP complex.^32,33^ In the cotranslational pathway, this complex is targeted to the inner membrane via interaction with the SRP receptor FtsY. Engagement of FtsY then facilitates transfer of the signal peptide to the SecYEG translocon.^32,33^ Upon docking to SecYEG, SRP and FtsY dissociate, and the amino-terminal signal peptide directly enters the translocon to initiate membrane insertion.^34^ Translation proceeds with the nascent chain being threaded into the channel, and hydrophobic α-helical transmembrane segments are laterally released from SecYEG into the lipid bilayer in a vectorial and stepwise manner. This tight coupling of translation and membrane insertion minimizes cytosolic exposure of hydrophobic segments and consequently reduces aggregation risk. Proteins destined for the inner membrane are predominantly targeted via this cotranslational pathway.^24,35,36^

In bacteria, two main machineries mediate the insertion of α-helical transmembrane proteins into the inner membrane: the SecYEG translocon and the YidC insertase. These systems can act independently or cooperatively during membrane protein biogenesis, and they may act either simultaneously or sequentially, depending on the substrate. The SecYEG translocon is a heterotrimeric transmembrane complex residing in the inner membrane composed of three subunits, SecY, SecE, and SecG.^37–39^ SecY forms the central protein-conducting channel, complemented by SecE and SecG forming the core translocation pore.^40–42^ In the cellular context, SecYEG is frequently embedded within a larger assembly termed the holo-translocon.^42^ In this complex, SecYEG associates with the trimeric accessory complex SecDF–YajC^43^ and the membrane insertase YidC,^44^ which enhance the efficiency of membrane protein insertion.^43,45^ Membrane insertion or translocation is initiated by engagement of the amino-terminal signal sequence with SecYEG. For inner membrane proteins, hydrophobic segments are laterally released into the lipid bilayer in a sequential manner, whereas secreted substrates traverse the channel.^46,47^ Thereby, cotranslational insertion is driven by ongoing translation, while post-translational translocation relies on the ATPase activity of SecA and further coupled to the proton-motive force by SecDF.^43,48,49^ SecDF is particularly important for the efficient translocation of large periplasmic loops and domains, where it promotes forward movement of the polypeptide through the SecYEG channel.

YidC functions as a monomeric inner membrane insertase that can operate either in conjunction with SecYEG or independently.^50,51^ Structurally, YidC forms a hydrophilic groove within the lipid bilayer that facilitates insertion and topological arrangement of α-helical transmembrane segments.^52,53^ It further contains a cytosolic domain implicated in substrate recognition and a periplasmic domain,^54,55^ which is thought to facilitate interactions with SecYEG.^55–58^ The YidC insertase belongs to a broadly conserved protein family that includes the mitochondrial Oxa1, the chloroplast Alb3, and evolutionarily distant homologues such as EMC3 and Get1 of the eukaryotic endoplasmic reticulum, as demonstrated by recent structural and functional studies.^59–62^ Although frequently associated with the holo-translocon, YidC can also mediate Sec-independent insertion of a subset of inner membrane proteins.^63^ Beyond its insertase activity, YidC has been shown to function as a molecular chaperone.^53,64^ Analogous to cytosolic chaperones that shield hydrophobic regions of nascent chains in the aqueous solution, YidC may protect marginally hydrophobic stretches of membrane protein precursors from the hydrophobic membrane environment, thereby preventing aggregation and promoting correct protein folding.^64,65^

The insertion of β-barrel proteins into the outer membrane of Gram-negative bacteria is mediated by the β-barrel assembly machinery (BAM) complex.^66^ The heteropentameric BAM complex consists of the central β-barrel protein BamA, which contains five periplasmic POTRA domains, and accessory lipoproteins BamB, BamC, BamD, and BamE.^67,68^ The POTRA domains of BamA mediate substrate recognition and interaction with the accessory factors, while the transmembrane domain of BamA is thought to catalyze folding and insertion of incoming outer membrane proteins.^69,70^ Importantly, in the native context, BAM complexes insert β-barrel proteins into a highly asymmetric membrane, with phospholipids in the inner leaflet and lipopolysaccharides (LPS) in the outer leaflet.^18,71^ This membrane is mechanically and chemically distinct from the inner membrane, exhibiting reduced lateral fluidity, which directly impacts the folding, insertion, and stability of β-barrel proteins.^72^ β-Barrel precursors are synthesized in the cytoplasm and translocated across the inner membrane into the periplasm via SecYEG.^73^ In the periplasm β-barrel precursors are captured and protected from the hydrophilic environment primarily by the holdase chaperone SurA.^33,23^ SurA, structurally related to TF, maintains incoming substrates in a dynamically unfolded folding-competent state and mediates their bulk transport to the BAM complex in the outer membrane.^74–76^ A secondary pathway involving Skp and the protease/chaperone DegP has been proposed^77^ but is thought to function primarily in quality control.^27,78^ Interestingly, recent studies challenge the classical view that SecYEG and BAM act independently. Instead, evidence points at physical and functional coupling between the inner membrane translocon and the BAM complex, potentially forming a periplasm-spanning supercomplex that coordinates substrate transfer across the envelope^79^ aided by chaperones including SurA and Skp.^66,77^

While cellular membrane protein biogenesis is orchestrated by a network of translocases, insertases, and chaperones, folding remains fundamentally constrained by the protein’s underlying energetic landscape. The free-energy landscape of protein folding resembles a funnel, with local (and occasionally global) minima corresponding to folding intermediates or misfolded states.^80^ Large membrane proteins frequently fold through multiple, coexisting pathways, reflecting the complexity of their energy landscapes and folding intermediates.^57,81^ How proteins navigate these pathways efficiently, and how the cellular machineries act on and reshape the folding landscape to ensure membrane protein maturation, remains largely unresolved. Resolving these pathways at the single-molecule level is crucial for understanding membrane protein biogenesis on a mechanistic level. However, since ensemble measurements average over diverse pathways and obscure transient intermediates, only a limited number of experimental techniques can investigate folding pathways under near-physiological conditions. Atomic force microscopy (AFM)-based single-molecule force spectroscopy (SMFS) provides such a window, allowing the direct probing of folding events and revealing how cellular machineries actively sculpt the folding energy landscape of single membrane proteins.

This review focuses on highlighting the cutting-edge AFM-based SMFS approaches that enable characterization of mechanistic aspects of insertase-, translocon-, and chaperone-assisted insertion and folding of membrane proteins at unprecedented molecular detail. Throughout, we focus on the Pf3 coat protein expressed in *E. coli* as a single α-helical transmembrane protein, the lactose permease LacY of the inner membrane of *E. coli* as a multispanning α-helical membrane protein, and the ferric hydroxamate uptake receptor (FhuA) of the outer membrane of *E. coli* as a complex β-barrel membrane protein. Although there are a few more examples for which the assisted insertion and folding into membranes have been studied using AFM-based approaches,^82^ these proteins serve as excellent model systems to illustrate the mechanistic insights, which can be gained through this unique nanotool.

## 2 Force-Based Methods to Study Membrane Protein Insertion and Folding

Mechanical force has emerged as a powerful tool for probing membrane protein unfolding and folding pathways at the single-molecule level. Several complementary force-based techniques have been introduced, including AFM-based single-molecule force spectroscopy (SMFS), optical and magnetic tweezers, steric trapping, and force-sensing translational arrest assays.^4,53,78,83–89^ Optical and magnetic tweezers allow the application of precisely controlled forces over extended time scales and have been used to monitor folding and unfolding transitions of membrane proteins reconstituted in lipid bilayers or nanodiscs. Steric trapping approaches use bulky ligands bound to engineered sites to mechanically destabilize folded states, enabling the thermodynamic stability and folding equilibria of membrane proteins to be quantified under near-native conditions. In parallel, *in vivo* force sensors based on translational arrest peptides such as SecM have provided detailed insights into cotranslational membrane protein insertion. In these assays, the pulling forces generated during membrane integration relieve ribosome stalling, allowing for the progression of translation to report on insertion energetics and topology formation with high spatial resolution. Collectively, these approaches complement each other by accessing different force regimes, time scales, and physiological contexts, thereby providing a broader view of the mechanical principles underlying membrane protein folding and insertion. However, this review concentrates on AFM-based SMFS approaches, as these methods are currently the most advanced for imaging and manipulating individual membrane proteins in synthetic or native membranes. In particular, they enable the detailed characterization and controlled perturbation of protein-specific unfolding and refolding pathways. Looking ahead, the complementary strengths of different force-based techniques may be integrated to provide a more comprehensive, multidimensional analysis of membrane protein folding and unfolding, capturing mechanistic features that are not accessible through a single method alone.

## 3 AFM-Based SMFS to Study Assisted Membrane Protein Insertion and Folding

AFM has become a widely used tool for imaging membrane proteins in the physiologically relevant environment, which is the lipid membrane, buffer solution, and ambient temperatures.^89,90^ Thereby, the AFM-based imaging can approach a lateral resolution ≤1 nm and a vertical resolution of ≈0.1–0.2 nm.^90–92^ However, the tip which the AFM uses to contour the topography of a membrane protein can at the same time also detect manyfold complementary physical, chemical, and biological information, such as mechanical properties, electrical fields, dynamic conformations and assemblies, ligand-binding, or interactions with viruses.^93,94^ As such AFM has developed into a nanoscopic multiparametric imaging tool that allows the characterization of membrane proteins at work.^94^ However, beyond imaging, AFM operated in the SMFS mode allows the mechanical probing of the structural stability and manipulate individual membrane proteins, providing detailed insights into their mechanical and thermodynamic stability, unfolding and folding pathways, multitude of inter- and intramolecular interactions, and conformational dynamics.^89,94–96^

SMFS experiments can be performed using nonspecific or site-specific strategies to attach membrane proteins to the SMFS probe, which in our case is the tip of the AFM cantilever. Site-specific approaches, including cysteine–maleimide chemistry, cysteine–gold coupling, sortase-mediated ligation, SpyTag/SpyCatcher, biotin–streptavidin, digoxigenin–antidigoxigenin, or Ni-NTA binding to His-tags, allow controlled orientation and improved reproducibility of unfolding measurements.^83,97,98^ However, such strategies can suffer from lower throughput, because once attached to the AFM tip the membrane protein will remain at the tip, where it can aggregate and contaminate the tip. Complementarily, nonspecific attachment, which generally avoids the need for site-specific genetic modifications, can benefit from engineering a polyG tail to extend the terminal end of a membrane protein, considerably enhancing the probability of attaching the terminal end of the protein to the tip of an AFM cantilever, while preserving the native state of the protein.^53,99,100^ Such an approach does not require chemically modifying the AFM tip, nor does it attach the membrane protein permanently to the tip. Consequently, the tip can be reused to repeatedly attach individual membrane proteins hundreds to thousand times, and thus to increase the experimental throughput.

In a typical AFM-SMFS experiment that relies on unspecific attachment, the tip of the AFM cantilever is brought into contact with membrane surface containing the membrane proteins of interest in buffer solution and ambient temperature. Thereby the cantilever is pushed onto the membrane protein at controlled force (commonly in the range of 0.5–1 nN; Figure 2a). This physical contact facilitates unspecific adhesion between the polypeptide of the membrane protein and the AFM tip via physisorption.^89^ In a probabilistic manner the AFM tip adheres unspecifically to one of the terminal ends of the membrane protein.^89^ Upon retraction of the cantilever, the mechanical force exerted on the polypeptide end induces the stepwise unfolding of the membrane protein, which is recorded in a force–distance curve, which displays a characteristic sawtooth pattern of force peaks (Figure 2b). Thereby, every force peak represents an unfolding step of a stable structural segment of the membrane protein. It has been shown that, depending on the sensitivity of the SMFS used, the individual force peaks can detect the unfolding of pairs or single transmembrane α-helices, β-strands, polypeptide loops, or even single helical turns of transmembrane α-helices.^97,101–105^ Together, all unfolding force peaks of the force–distance curve describe the sequential unfolding of the stable structural segments established by the membrane protein. Thereby, the magnitude and distance of each force peak correspond to the force needed to unfold the structural segments of the membrane protein and the extension of the polypeptide chain, respectively. If no protein attaches to the AFM tip, the recorded force–distance curve shows only baseline noise and lacks force peaks.

**Figure 2:**
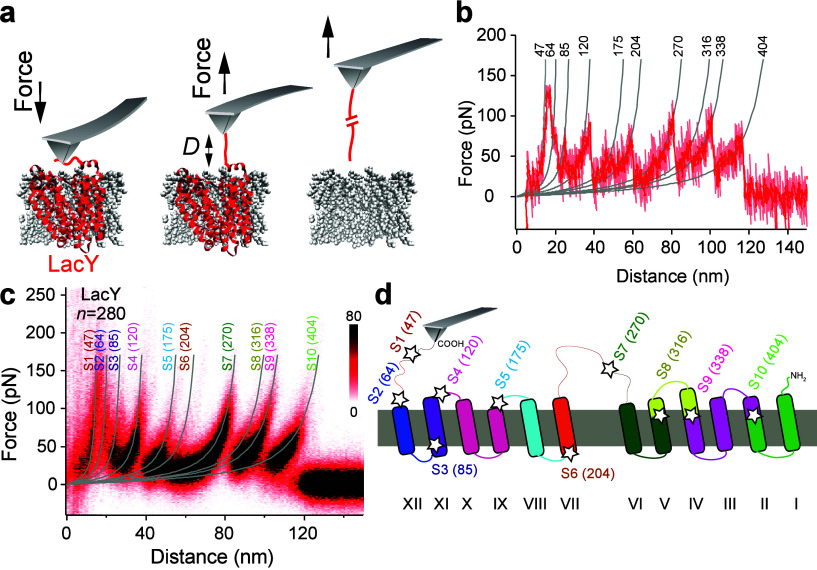
Mechanical unfolding fingerprint of native LacY. (a) Schematic of mechanically unfolding and extracting a native LacY from a phospholipid membrane (POPE:POPG) using AFM-based SMFS. The tip of the AFM cantilever is pressed onto LacY (PDB: 1PV7), enabling nonspecific attachment at the extended C-terminus (polyGly-LacY). Retraction of the cantilever applies a mechanical force that unfolds LacY in a stepwise manner until being fully extracted from the membrane. (b) Force–distance curve from the unfolding of a single LacY. Raw data (pale red) and a smoothed trace (Savitzky-Golay filter, dark red) are shown. Each force peak is fitted with the worm-like chain (WLC) model^89,97^ (gray curves) to calculate contour length of the unfolded polypeptide segment (in amino acids on the top of each WLC curve). (c) Density plot of 280 superimposed force–distance curves, each recording the mechanical unfolding of a single LacY. (d) Structural segments S1–S10 of LacY that are detected upon mechanical unfolding and mapped to the LacY secondary structure. Transmembrane α-helices are labeled I–XII. Reproduced with permission from ref.^57^ Copyright 2019 The American Association for the Advancement of Science.

At best scenario, the AFM tip unspecifically attaches to the terminal end of the membrane protein. However, it can also, with certain probability, attach to polypeptide loops connecting transmembrane α-helices or other polypeptide regions. In such cases, the resulting unfolding events, which are induced upon applying a mechanical force by the retracting AFM tip, produce force–distance curves, which are significantly shorter than those corresponding to the full unfolding and extension of the polypeptide of a membrane protein attaching with one of its terminal ends to the AFM tip. For analysis, these different attachment scenarios can be separated by selecting force–distance curves, which force peaks extend over distances (contour lengths) that correspond to the complete unfolding and mechanical stretching of the membrane protein.^89,97,106,107^ The superimposition of force–distance curves recorded upon fully unfolding multiple membrane proteins from the terminal end reveals reproducible force peak patterns, which are characteristic of the specific membrane protein and its folding state (Figure 2c).^53,97,99,102^

To quantitatively interpret force–distance curves that have been recorded upon mechanically unfolding of a single membrane protein, the worm-like chain (WLC) model of polymer elasticity is commonly applied (gray curves, Figure 2b,c).^89,99,108–110^ This approach assumes that the unfolded polypeptide chain of the protein behaves as an entropic polymer. Being fit to individual force peaks, the WLC model can then estimate the contour lengths of the polypeptide stretches that the membrane protein stepwise unfolds upon exposure to mechanical force. The contour length of each force peak can be used to locate the stable structural segment of the membrane protein that has resisted unfolding until the externally applied force has overcome its stability and the segment unfolded (Figure 2c,d). The localization of each structural segment within the membrane protein structure is typically done after some alignment.^106,111–113^ Together, all sequentially unfolded stable structural segments describe the unfolding pathway of the membrane protein. For lactose permease LacY, for example, such analysis has identified ten stable structural segments, each contributing to the stability of the unfolding membrane protein.^99^ Typically, such structural segments are represented by entire or parts of transmembrane α-helices and polypeptide loops. Notably, the positions of the unfolding intermediates do not strictly coincide with transmembrane α-helical boundaries and stabilizing structural segments can locate within α-helices or polypeptide loops. This likely reflects the presence of local structural features, such as α-helical kinks, partially uncoiled α-helical regions, or short structured polypeptide loops, which can introduce mechanical barriers along the unfolding pathway. Together the stable structural segments detected by AFM-based SMFS provide at the resolution of secondary structural elements the individual steps, or unfolding intermediates, a membrane protein takes along its unfolding pathway.

Applying this SMFS principle, as illustrated with the example of LacY (Figure 2), to other membrane proteins has enabled extensive characterization of how environmental factors such as temperature, pH, electrolyte, lipid composition, ligand binding, or point mutations structurally stabilize or destabilize membrane proteins.^99,107,114–121^ Consequently, SMFS can contribute to understanding the fundamental principles governing membrane protein stability and folding as well as the mechanisms leading to their malfunction.

## 4 Lipid-Dependent Alterations in Membrane Protein Folding

The lipid environment plays a critical role in the stability, folding, and conformation of membrane proteins.^109,117,118,122^ SMFS has the sensitivity to directly probe how lipids and lipid compositions alter the membrane protein stability.^108,117,118,121^ On the example of LacY, SMFS revealed that lipid compositions can alter the overall stability and fold of the membrane protein (Figures 2 and 3). In this study, LacY was investigated in two lipid environments represented by a 3:1 mixture of POPE and POPG, which mimics the physiological membrane composition, and by solely POPG. SMFS was used to mechanically unfold LacY from both lipid membranes (Figure 2 and Figure 3). In POPE:POPG membranes, the force–distance curves detected one characteristic force peak pattern corresponding to that of native LacY (Figure 3a, Figure 2). This force peak pattern hence characterized the mechanical unfolding pathway of native LacY. Interestingly, however, in the POPG membrane, SMFS detected two classes of force–distance curves upon unfolding LacY. The second class of force–distance curves did not resemble the first force peak pattern and thus the unfolding pattern of native LacY (Figure 3b). Superimposition of the force–distance curves of the second class obtained in the POPG-only condition showed an increased noise of the force and that some force peaks shifted or/and additional force peaks occurred (Figure 3c,d). These observations indicate considerable conformational heterogeneity of LacY and support the previously proposed hypothesis that the imbalance in the ratio of anionic and zwitterionic lipids can promote a non-native, nonfunctional conformation of LacY.^123,124^

**Figure 3:**
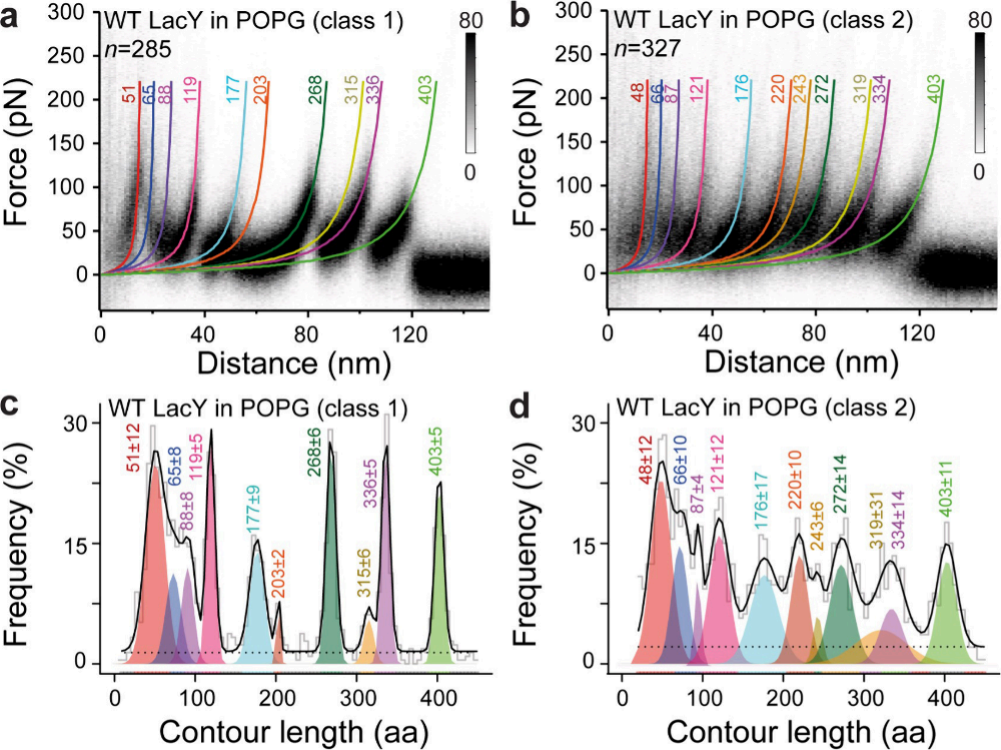
SMFS detects two classes of unfolding patterns of LacY in the POPG membrane. (a, b) Density plots of superimposed force–distance curves, which were recorded upon the mechanical extraction and unfolding of LacY from POPG membranes. The plots show two classes (1 and 2) of force peak patterns. Colored WLC fits indicate mean contour lengths of the individual force peaks. *n* indicates the number of force–distance curves analyzed. (c, d) Contour length histograms for class 1 (c) and class 2 (d) force peak patterns, fitted using a Gaussian mixture model. Ten and 11 force peaks were identified in class 1 and class 2, respectively. Colored peaks and WLC curves show mean contour lengths (±SD) of the force peaks. Black lines indicate the total fit; dashed lines show baseline noise. Reproduced with permission from ref.^121^ Copyright 2015 Elsevier.

LacY forms 10 stable structural segments in POPE:POPG, but 11 in POPG membrane (Figure 2, Figure 3). The newly appearing force peaks observed in the 220–250 amino acid region, which encompasses transmembrane α-helices VI and VII, indicate structural aberrations in this structural region of LacY (Figure 4). Biochemical investigations performed in bulk indicate that transmembrane α-helix VII changes topology in response to the negatively charged POPG.^123–125^ In particular, previous studies suggested that the marginally hydrophobic transmembrane α-helix VII of LacY becomes periplasmic if exposed to a POPG membrane and the N terminal half of the LacY protein inverts its topology^123–125^ (Figure 4). This hypothesis was generated based on LacY loops accessibility analysis.^123^ The SMFS data directly detects that α-helix VII in POPG membranes folds differently, while the entire LacY structure is destabilized.

**Figure 4:**
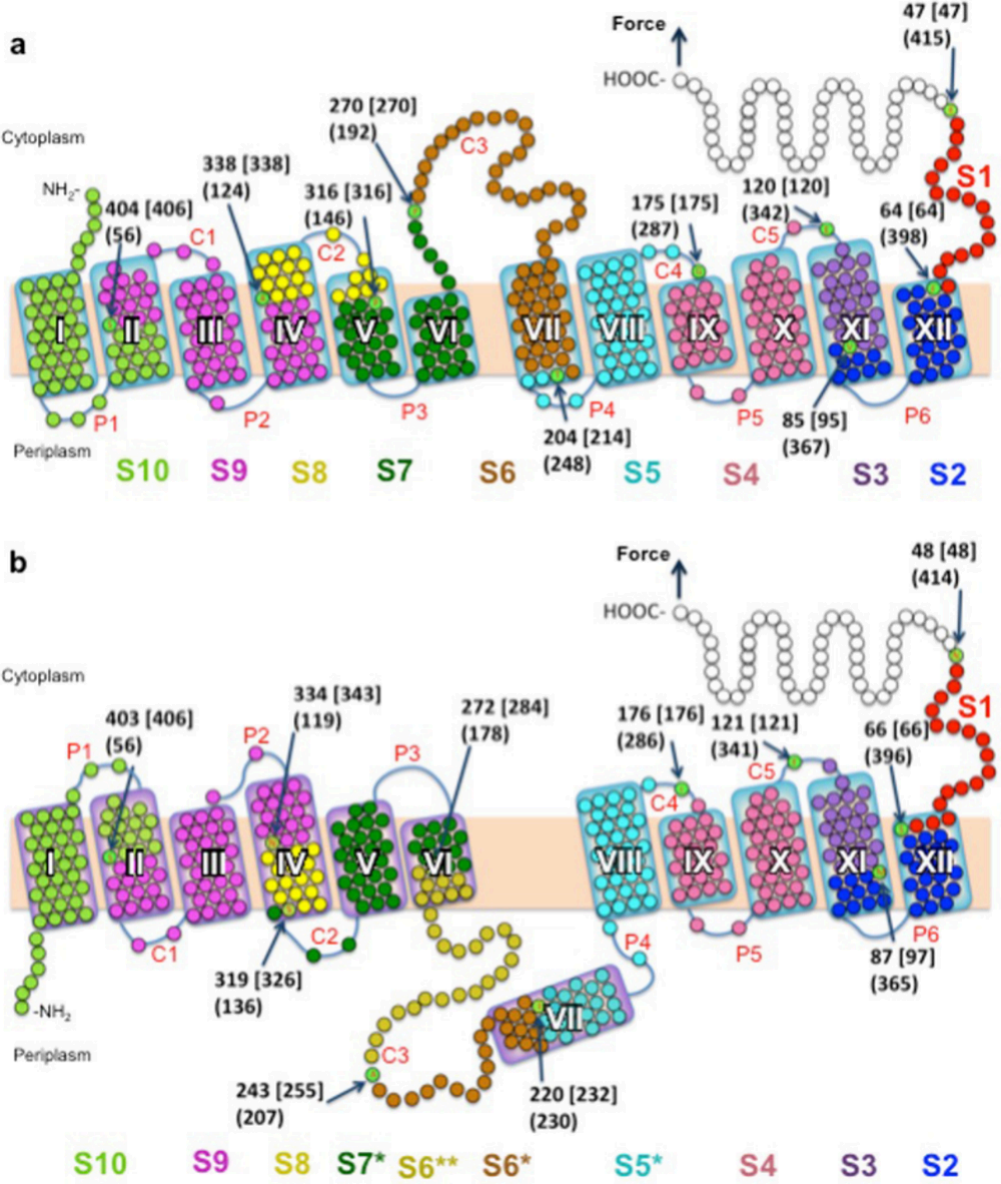
Effect of lipid composition on the folding of LacY. (a, b) Structural segments mapped to the secondary structure of WT LacY adopting the native (a) and the inverted (b) topology in POPE:POPG and POPG lipid membranes, respectively. Stable structural segments mapped in (a) and (b) were detected in the class 1 and class 2 force–distance curves, respectively. (a) Secondary structure model of LacY (PDB: 1PV7) mapped with 10 (class 1 force–distance curves) structural segments S1–S10 (native topology) and (b) with 11 (class 2 force–distance curves) structural segments S1–S4, S5*, S6*, S6**, S7*, S8, S9, S10 (inverted topology). Each mean contour length of a force peak class assigns the beginning of a structural segment (arrows pointing to aa). The numbers at arrows show the mean contour lengths of a force peak class (in aa), numbers in square brackets indicate the aa position counted from the C-terminal end, and numbers in parentheses give the aa position from the N-terminal end. Each of these numbers distinguishes the end of the previous and the beginning of a stable structural segment. Reproduced with permission from ref.^121^ Copyright 2015 Elsevier.

The quantitative analysis of the force–distance curves reveal that ≈50% of LacY in POPG-only membranes adopt non-native conformations.^121^ Such quantifications are hardly possible with bulk methods that average signal over all co-occurring topologies. Interestingly, the detailed analysis of LacY topology in POPE:POPG (3:1) membranes reveals that also in this native-like lipid composition a small fraction of LacY (5%) adopts the non-native topology.^121^ In terms of free-energy landscape, this suggest that both states of LacY, native and non-native, are approximately equally favorable in the POPG membrane, while the native state is much more thermodynamically favorable in the POPE:POPG (3:1) membrane. Together, these findings underscore the impact of the membrane lipid composition on the folding landscape of LacY and highlight the enormous sensitivity of SMFS to resolve such effects at the single-molecule level. More broadly, they illustrate that SMFS can be applied to characterize how lipid–protein interactions modulate the membrane protein structure and function.

## 5 Assisted Insertion and Folding of α-Helical Membrane Proteins

Membrane proteins can insert into membranes through multiple pathways. In cells, many α-helical membrane proteins are integrated cotranslationally via the signal recognition particle (SRP)-dependent SecYEG translocon, whereas membrane insertases such as YidC can mediate insertion either independently or in cooperation with SecYEG, often together with additional accessory factors. Before considering assisted insertion pathways, it is important to note that some membrane proteins can spontaneously insert and fold into lipid bilayers, without the aid of translocons or insertases.^11,126^ Such behavior has been demonstrated not only by AFM-based SMFS studies but also by biochemical and biophysical experiments and molecular dynamics simulations.^126–128^ The propensity for spontaneous insertion depends strongly on physicochemical properties of the protein, including the hydrophobicity and length of transmembrane segments, the distribution of charged and polar residues, the size and topology of extramembrane domains, and the properties of the lipid membrane itself. Thermodynamically, the insertion is driven by favorable partitioning of hydrophobic regions into the membrane, whereas large hydrophilic domains or complex topologies can impose kinetic and energetic barriers. In particular, many AFM-based SMFS studies of the spontaneous insertion of membrane proteins have been performed after partial unfolding or extraction of a membrane protein, which may lower the insertion and folding barriers. This behavior has been most clearly demonstrated for relatively small membrane proteins, including small β-barrel membrane proteins, and may not be directly generalizable to larger or more complex systems. The spontaneous insertion of membrane proteins into lipid membranes has been demonstrated *in vitro*. However, whether membrane proteins can insert entirely without assistance *in vivo* remains an open question, as even membrane proteins that are independent of the Sec pathway may rely on alternative machineries that reduce the energetic barriers to membrane integration (*e.g*., YidC). Consequently, while certain membrane proteins or domains can insert autonomously under favorable conditions, most multispanning membrane proteins require dedicated systems such as SecYEG and YidC for efficient and accurate biogenesis.

Despite being extensively studied, fundamental questions remain regarding how membrane proteins are inserted and folded into membranes. These include how nascent transmembrane segments initially engage with the membrane, how correct topology is established and maintained, how polypeptide loops translocate across the membrane, and how proteins are protected from misfolding or aggregation during biogenesis. Additional open issues concern the energetic contributions of lipid composition, the structural basis of substrate recognition by insertases, and the mechanisms by which SecYEG and YidC function together during insertion and folding.

AFM-based SMFS provides a complementary approach to traditional ensemble techniques by enabling the direct observation of insertion, unfolding, and refolding of individual proteins in defined membrane environments at the resolution of individual stable structural segments. This capability allows individual insertion steps, transient intermediates, and topology changes to be monitored directly, thereby offering unique insights into how membrane proteins interact with lipid bilayers and insertion machinery, how structural segments assemble, and how misfolding is prevented during biogenesis. By probing these processes at the single-molecule level, SMFS can address many of the unresolved questions outlined above.

### 5.1 Binding and Insertion of Single Domain Membrane Proteins by YidC

YidC functions as both an insertase and a molecular chaperone, facilitating the folding of a wide range of membrane proteins.^129–131^ To investigate how YidC promotes membrane protein folding at the structural level, YidC was reconstituted into POPE:POPG membranes and various AFM-based SMFS modes were applied to monitor how YidC assists the folding of both short and large, multispanning membrane proteins.

To study the YidC-assisted insertion and folding of small single-spanning transmembrane proteins, Pf3, a short polypeptide, was probed using AFM-based SMFS.^58^ After specific chemical attachment of the Pf3 polypeptide to the AFM tip, the AFM cantilever was used to bring the Pf3 polypeptide in close proximity with a POPE:POPG (3:1) phospholipid membrane containing embedded YidC. Force–distance curves showed characteristic force peaks indicating that Pf3 could bind to YidC. Control experiments using phospholipid membranes in the absence of YidC showed no such interactions. Moreover, by varying the contact time between the Pf3 and YidC, it became possible to reveal mechanistic insights into how YidC binds and inserts short transmembrane proteins. Pf3 bound to the cytoplasmic hairpin of YidC within a few milliseconds, while longer contact times enabled membrane insertion. Time-dependent SMFS measurements revealed distinct force peaks that reflect the force of the Pf3–YidC interaction and the force needed to extract and unfold Pf3 from the membrane. Together with molecular dynamics (MD) simulations, these results supported a two-step mechanism through which YidC first binds and then inserts and folds Pf3 into the membrane.

### 5.2 Insertion of Multispanning Membrane Proteins by YidC

To determine how YidC inserts and folds large transmembrane proteins, both YidC and the target membrane protein were coreconstituted into the same lipid membrane. This approach allowed, for example, both LacY and YidC to be incorporated and distributed within the same POPE:POPG (3:1) lipid membrane.^53^ It was observed that in these membranes LacY and YidC resided in close proximity to each other.^53^ AFM-based SMFS was used to image these membranes, and afterward a single LacY was mechanically partially unfolded while leaving the last stable structural segment embedded in the membrane. This partially unfolded LacY polypeptide was then mechanically relaxed to characterize its insertion and folding into the membrane in the presence of YidC (Figure 5). After a certain folding time has passed, the LacY polypeptide was unfolded again to observe which structural segments had misfolded or folded. Upon characterizing how the partially unfolded LacY polypeptide inserts and folds within a range of insertion and folding times, the experiments could characterize which structural segments of LacY self-inserted and folded faster or slower and which segments were prone to misfolding.

**Figure 5:**
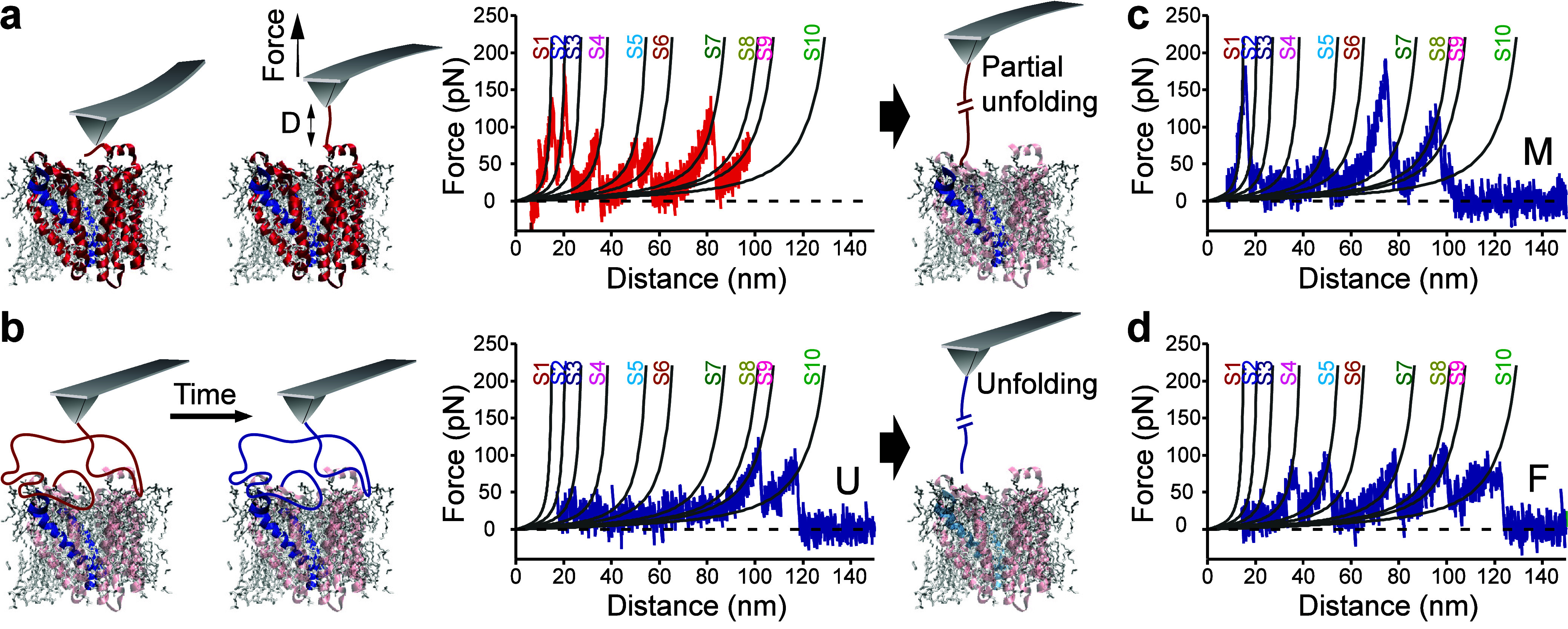
Single-molecule insertion and refolding of partially unfolded LacY into the phospholipid membrane. (a) Schematic of the refolding experiment of a single LacY using AFM-based SMFS. The tip of the AFM cantilever is attached to the C-terminus of LacY, and the cantilever is retracted to sequentially unfold the protein, leaving the last stable structural segment (S10, blue) embedded in the membrane. The resulting force–distance curve reflects the unfolding peaks of native LacY (see Figure 2). (b) The AFM tip is then brought close to the membrane (∼10 nm) to relax the unfolded polypeptide. After 2 s of refolding time, the AFM tip is fully retracted to assess whether LacY remained unfolded, misfolded, or refolded structural segments. (b–d) Representative force–distance curves for LacY that remained unfolded (b), misfolded (c), or refolded partially (d). All experiments were performed in 50 mM KPi buffer, pH 7.2, at 25 °C. Reproduced with permission from ref.^53^ Copyright 2016 Springer Nature.

To differentiate among correctly folded, misfolded, and unfolded structural segments, it is necessary to identify the characteristic force peak pattern of natively folded LacY, which serves as a fingerprint template. On the basis of this template, force peaks detected in refolding experiments allow the categorization of the unfolded LacY polypeptide as (*i*) unfolded, if no force peak appears in the previously unfolded portion of LacY thus indicating the lack of refolding, (*ii*) misfolded, if force peaks appear, which do not correspond those detected in the characteristic unfolding fingerprint pattern of the protein, thus indicating incorrect or incomplete folding, and (*iii*) refolded, if the force peaks appear only at positions that match those of the native unfolding fingerprint pattern, thus indicating correct folding. Refolding can be partial or complete, depending on whether a few or all characteristic force peaks were detected at the position of the force peaks in the native unfolding fingerprint pattern.

Following the above-described scheme, the refolding of the previously partially unfolded LacY was investigated in the presence and in the absence of YidC.^53^ Approximately 100 refolding events were analyzed for both conditions after a 2 s refolding time. Unfolding, misfolding, and folding events were detected. However, their probability differed significantly depending on the presence and absence of YidC (Figures 5 and 6). In the absence of YidC, partially unfolded LacY could refold some structural segments, but the probability of misfolding was high with 41% (Figure 6a). The presence of YidC reduced the fraction of LacY misfolding events considerably to 12% (Figure 6b). YidC also increased the fraction of force–distance curves corresponding to partial correct folding of LacY from 53% to 76%. Controls using nonspecific proteins such as bovine serum albumin (BSA) and lysozyme instead of YidC showed no significant impact on refolding outcomes.^53^ Altogether, the presence of YidC in the lipid membrane considerably increased the proportion of correctly folded LacY molecules while the fraction of misfolding events was reduced (Figure 6). The same tendency was observed for the individual structural segments of LacY (Figure 6c,d).

**Figure 6:**
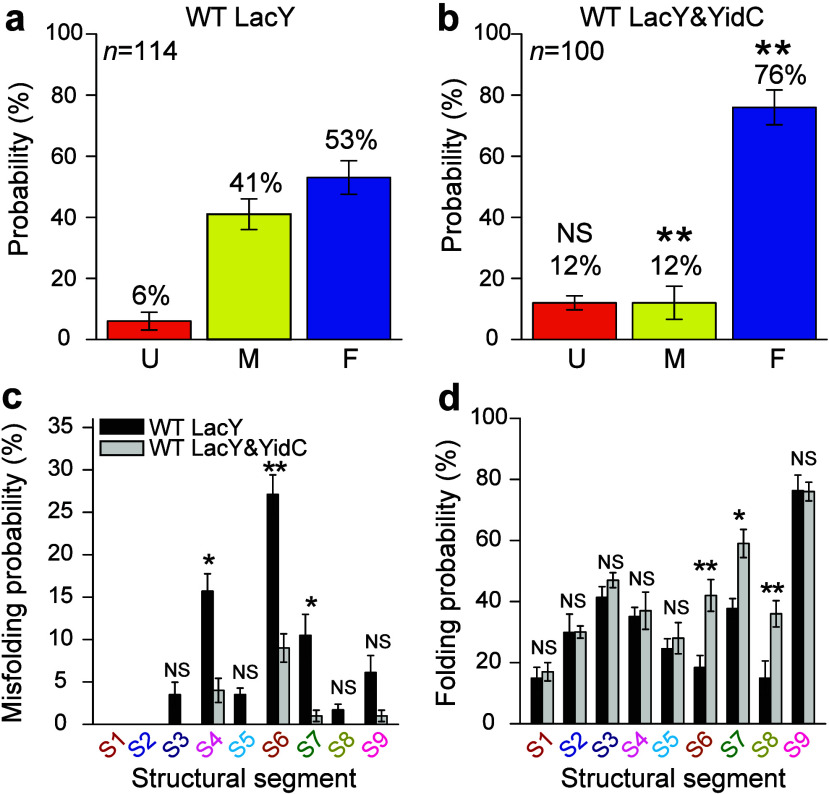
YidC suppresses misfolding and promotes proper insertion and folding of LacY into the phospholipid membrane. (a, b) Probability of unfolded LacY remaining unfolded (U), having misfolded (M), or having correctly refolded (F) in the absence (a) or presence (b) of YidC. (c) Probability of structural segments S1–S9 to misfold in the presence and absence of YidC. (d) Probability of proper insertion and folding of the structural segments under the same conditions. Differences of refolding experiments conducted in the absence and presence of YidC are considered significant. Significance tests with respect to WT LacY data are indicated: NS, *P* ≥ 0.01, * *P* < 0.01 and ** *P* < 0.001 as determined by two-tailed Z-test. Adapted with permission from ref.^53^ Copyright 2016 Springer Nature.

Furthermore, these refolding experiments highlight that the structural segments S6, S4, and S7 of LacY are particularly prone to misfolding (Figure 6c). Segment S6 corresponds to transmembrane α-helix VII, which is characterized by its marginal hydrophobicity. This structural region of LacY, which is also primary affected by changes in lipid composition (Figures 3 and 4), is suggested to be an “Achilles’ heel” of LacY.^53,121,123–125^ Segment S6 also contains negatively charged residues (Asp237, Asp240) that form salt bridges with lysine residues in transmembrane α-helices X and XI (segment S4). Segment S7 connects to S6 via a cytoplasmic loop and therefore might also be linked to the misfolding of S6 (Figure 6c). Therefore, the preferential misfolding of these three regions in LacY is likely the result of a misfolding-cascade, initiated by misfolding of transmembrane α-helix VII. The chaperone activity of YidC notably prevented misfolding and improved folding in these regions (Figure 6c,d).

The results also suggested that a 2 s refolding time is insufficient for LacY to complete its insertion and folding. Analysis across refolding times from 0.1 to 5 s revealed that the number of refolded LacY segments increased in the presence of YidC, indicating that YidC assists in the stepwise insertion and folding of structural segments into the membrane. Individual analysis of these refolding events showed no preferred order of insertion, suggesting that LacY segments insert and refold in a stochastic order.

Together, these findings suggest that YidC promotes the stepwise and stochastic refolding of LacY segments into the lipid membrane by providing multiple possible coexisting folding pathways (Figure 7). In terms of the free-energy landscape, YidC increases the free-energy barrier toward misfolding, which otherwise prevents the correct folding of LacY to complete, thereby funneling the protein to the correct fold.

**Figure 7:**
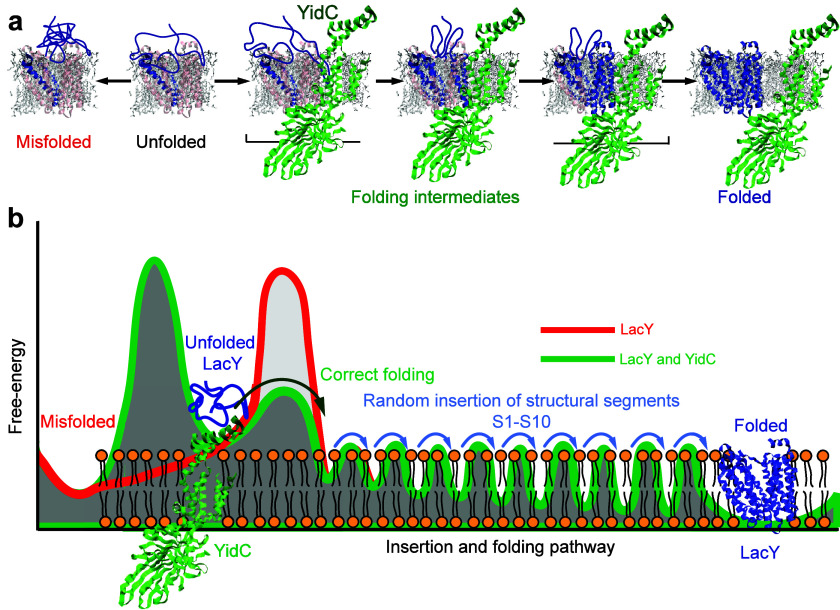
Model of the folding free-energy landscape of partially unfolded LacY in the presence of YidC. (a) In the absence of YidC, partially unfolded LacY misfolds with high probability. YidC prevents this misfolding and directs the partially unfolded LacY substrate through multiple folding intermediates toward folding its native state. (b) In the absence of YidC, the partially unfolded LacY inserts individual structural segments (S1–S10) into the membrane (red profile). However, folding is not completed and the majority of LacY substrates misfold after 5 s. YidC reduces misfolding and allows the unfolded LacY substrate to stepwise insert and fold its structural segments (S1–S10) into the membrane (green profile). Thereby, the structural segments insert and fold in random order until the folding of LacY has been completed. Adapted with permissions from refs. 53,132 Copyright 2016 Springer Nature and copyright 2017 American Chemical Society.

### 5.3 Insertion of Multispanning Membrane Proteins by EMC

Recent work has extended force-based approaches to investigate the insertase-assisted folding of complex eukaryotic membrane proteins.^83^ For example, magnetic tweezer experiments have been used to monitor the refolding of the multispanning glucose transporter GLUT3 in the presence and absence of the endoplasmic reticulum membrane protein complex (EMC), an insertase implicated in the biogenesis of many eukaryotic membrane proteins. In this assay, a single unfolded GLUT3 molecule tethered between a magnetic bead and a surface was allowed to refold under a constant force while embedded in a lipid membrane. The presence of EMC facilitated the insertion of GLUT3, demonstrating that EMC supports the folding of marginally hydrophobic regions and lowers the energy barriers associated with membrane insertion. However, it was also observed that folding proceeds asymmetrically, with the N-terminal domain forming first and thereafter guiding the assembly of the more-challenging C-terminal domain. Complementary to the observations made from AFM-based studies of bacterial insertases such as YidC, these findings suggest that diverse insertases across kingdoms facilitate membrane protein biogenesis by reshaping the folding energy landscape and stabilizing marginally hydrophobic regions. Notably, the completion of the membrane protein domain assembly further depends on specific lipid interactions that promote a proper packing within the membrane. Importantly, magnetic tweezers provide continuous, long-duration measurements under constant force, complementing the stepwise unfolding and folding information obtained from the AFM-based SMFS. Together, these two force-based methodologies offer a convergent view of how mechanical forces and cellular machinery cooperate to guide membrane protein insertion and folding in membranes.

### 5.4 AFM-Based Pull-and-Paste Assay of Single Membrane Proteins

In addition to enabling studies on membrane proteins that have been partially unfolded and extracted from the membrane, AFM-based SMFS can be employed to investigate the insertion and folding of completely unfolded membrane proteins at the single-molecule level. The “pull-and-paste” assay utilizes the AFM tip to extract and unfold a single membrane protein from a reservoir membrane and to transfer the unfolded polypeptide to lipid membranes containing the insertase YidC (Figure 8).^132^ To apply this assay, proteoliposomes containing the donor membrane protein (e.g., LacY) and target membranes containing, for example, YidC are coadsorbed onto a substrate and identified by AFM (Figures 8, 9a).^89^ Then, using the AFM tip, a single LacY is mechanically completely unfolded and extracted from a donor membrane using the AFM tip (Figure 9b) and transferred to the target membrane containing YidC. The unfolded polypeptide is held close to the target membrane for a defined time to allow insertion and folding. After this insertion and folding time passed, AFM-based SMFS is used to characterize whether the LacY polypeptide inserted and folded into the target membrane.

**Figure 8:**
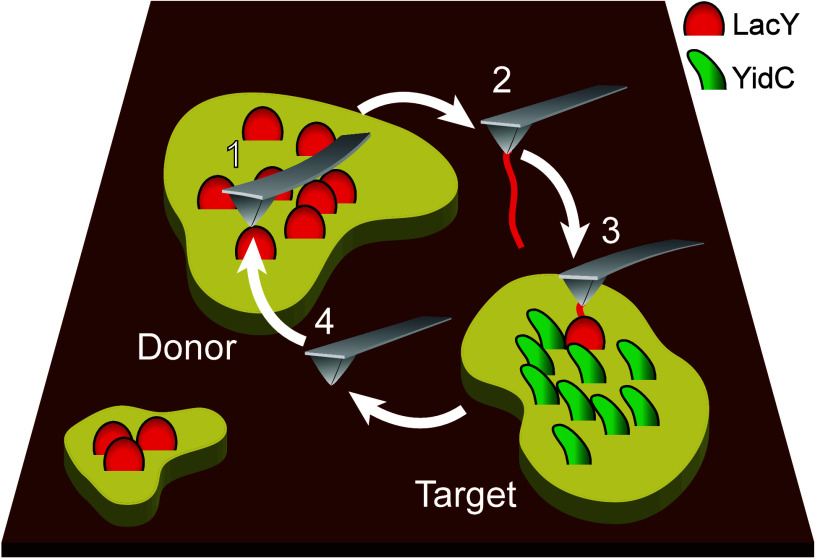
Schematic of the pull-and-paste assay to study the insertion and folding of single transmembrane proteins into a target membrane. The C-terminal end of a LacY (red) from a donor membrane is attached unspecifically to the tip of an AFM cantilever. Then, the LacY polypeptide is mechanically extracted and unfolded from the phospholipid membrane (1). The unfolded polypeptide is transferred to a target membrane containing the chaperone/insertase YidC (green) (2). At the YidC, the unfolded polypeptide is allowed to insert and fold into the membrane for a given time (3). With time, the unfolded LacY polypeptide progressively inserts and folds into the target membrane, ultimately adopting its native structure. Due to the temporary attachment of the C-terminal end to the tip, the end eventually slips off, allowing the tip to pick up another LacY for mechanical reconstitution (4). Reproduced with permission from ref.^132^ Copyright 2017 American Chemical Society.

**Figure 9:**
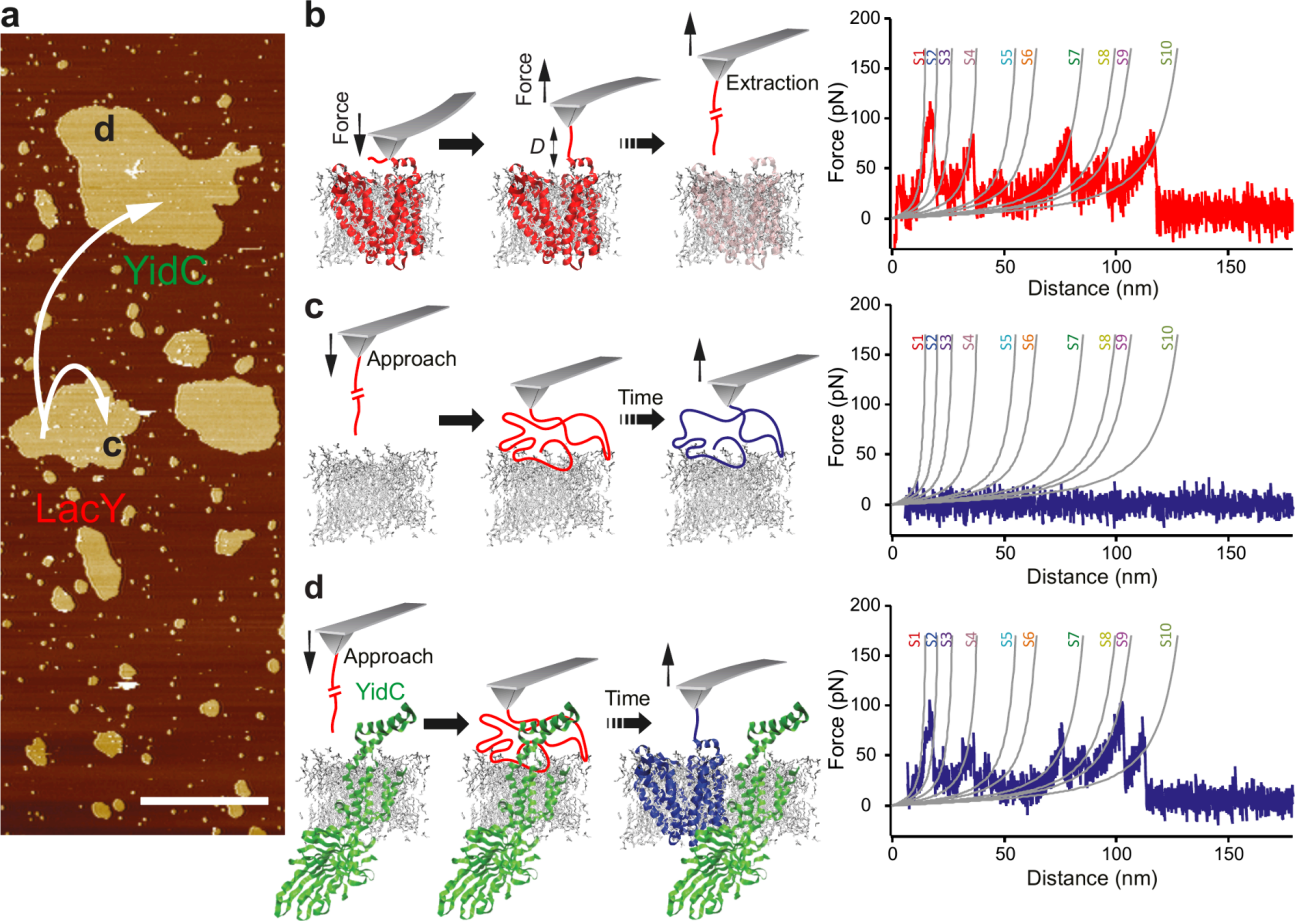
YidC facilitates the integration and folding of extracted, unfolded LacY into the membrane. (a) AFM height image (topography) showing membranes containing LacY and YidC on mica in buffer solution. Scale bar: 1 μm. (b) Schematic of LacY (red) unfolding and extraction from a membrane by AFM-based SMFS. The AFM tip is first attached to the C-terminus, then the tip is retracted to mechanically extract and stepwise unfold the protein from the membrane. The resulting force–distance curve (red) shows characteristic unfolding peaks (segments S1–S10), matching the unfolding peaks recorded of native LacY (see Figure 2). Gray WLC fits highlight each unfolding peak and step of LacY. (c) After extraction, the unfolded LacY polypeptide is laterally shifted 20–100 nm and held near (≈10 nm) a control membrane for 5 s. No insertion and refolding into the membrane is observed upon retraction. (d) In contrast, when LacY is moved to a YidC-containing membrane and held near the surface for 5 s, the force–distance curve (blue) shows native-like unfolding peaks, indicating the successful reinsertion and folding of the polypeptide into the native LacY structure. Reproduced with permission from ref.^132^ Copyright 2017 American Chemical Society.

Control experiments show that the unfolded LacY polypeptide does not insert and fold when it is held close to a membrane lacking YidC (Figure 9c).^132^ In contrast, the presence of YidC enables the refolding of LacY into the membrane (Figure 9d), demonstrating that YidC alone is sufficient to mediate the insertion and folding of the completely unfolded polypeptide into the membrane. Notably, it was observed that folding could be initiated from any structural segment of LacY, highlighting the flexibility of YidC in initiating the membrane insertion and folding process.^132^ Upon extension of the folding time, more structural segments inserted into the membrane, indicating the stepwise insertion and folding of the unfolded LacY polypeptide. Interestingly, the sequence of insertion and folding events appeared random, consistent with observations made for partially unfolded LacY.^53^ At prolonged refolding times of 5 s, the LacY polypeptide was able to complete its insertion and folding process in the presence of YidC.

These findings collectively suggest that, in the absence of the insertase YidC, the unfolded LacY is unable to initiate folding due to the presence of a too high energy barrier that precludes spontaneous membrane insertion. YidC, however, lowers this barrier and chaperones membrane insertion and folding of LacY until completion. Mechanistically, this chaperoning of the folding process is thought to be facilitated by a hydrophilic groove located within the core of YidC.^54,55^ This groove, in conjunction with a conserved positively charged arginine residue (Arg 366) of YidC, may stabilize and guide marginally hydrophobic and negatively charged segments of the protein during its insertion. Additionally, local membrane thinning in the vicinity of YidC may further contribute to the reduction of the energetic barrier to insertion.^133^

Generally, the pull-and-paste assay allows a single membrane protein to be mechanically extracted and unfolded from a donor membrane and studied for its insertion and folding process into a different target membrane. Because the unfolded membrane protein is mechanically delivered directly to the target membrane, the assay minimizes the prior perturbation of the donor membrane, which could be associated with the mechanical extraction procedure. The assay also provides a controlled way to reconstitute specific proteins into membranes, which may be useful for future biotechnological applications and for designing membranes with defined protein compositions.

### 5.5 Insertion of Multispanning Membrane Proteins by SecYEG Alone and in Tandem with YidC

SMFS-based folding assays are not limited to studying the function of the YidC insertase. In principle, any insertase or translocase can be characterized using the pull-and-paste assay of membrane proteins. One prominent example is the SecYEG translocon, a structurally and mechanistically distinct membrane protein translocase. Structurally, YidC and SecYEG differ markedly: SecYEG contains a central channel and a lateral gate that facilitate the release of structural segments into the lipid membrane.^54,134–136^ SecYEG inserts α-helical inner membrane proteins, which are characterized by long hydrophobic sequence stretches, and translocates periplasmic and β-barrel membrane protein precursors across the inner membrane.^137,138^

For example, the SecYEG translocon was reconstituted into lipid membranes, which were coadsorbed as target membranes together with LacY-containing donor membranes, similar to that exemplified above. Using this setup, individual LacY molecules were first mechanically unfolded and extracted from donor membranes and afterward transferred to target membranes to characterize their insertion and folding assisted by SecYEG.^57^ Using this assay, it was observed that SecYEG can initiate insertion from any structural segment of LacY, highlighting the inherent flexibility of the translocon in engaging substrate polypeptides.^57^ In particular, even under these relaxed, post-translational conditions, structural segments inserted sequentially, indicating that the sequential integration of structural segments into the membrane is intrinsically encoded in the SecYEG machinery. This assay observed how SecYEG mediates the insertion of LacY, again starting from any structural segment, indicating great flexibility in initiating the insertion and folding process. This feature of allowing polypeptides to flexibly insert and fold structural segments into membranes appears to be a common characteristic of YidC and SecYEG. However, the overall kinetics of the SecYEG-assisted insertion and folding of structural segments of LacY were slower compared to the YidC insertase (Figure 10). For instance, the insertion and folding of LacY in the presence of SecYEG were completed within 10 s (Figures 10a and 10b), whereas YidC completed the insertion and folding of the LacY polypeptide within 5 s. Detailed kinetics analysis of these studies shows that the YidC-assisted insertion and folding of LacY are approximately twice as fast compared to SecYEG (Figure 10c).

**Figure 10:**
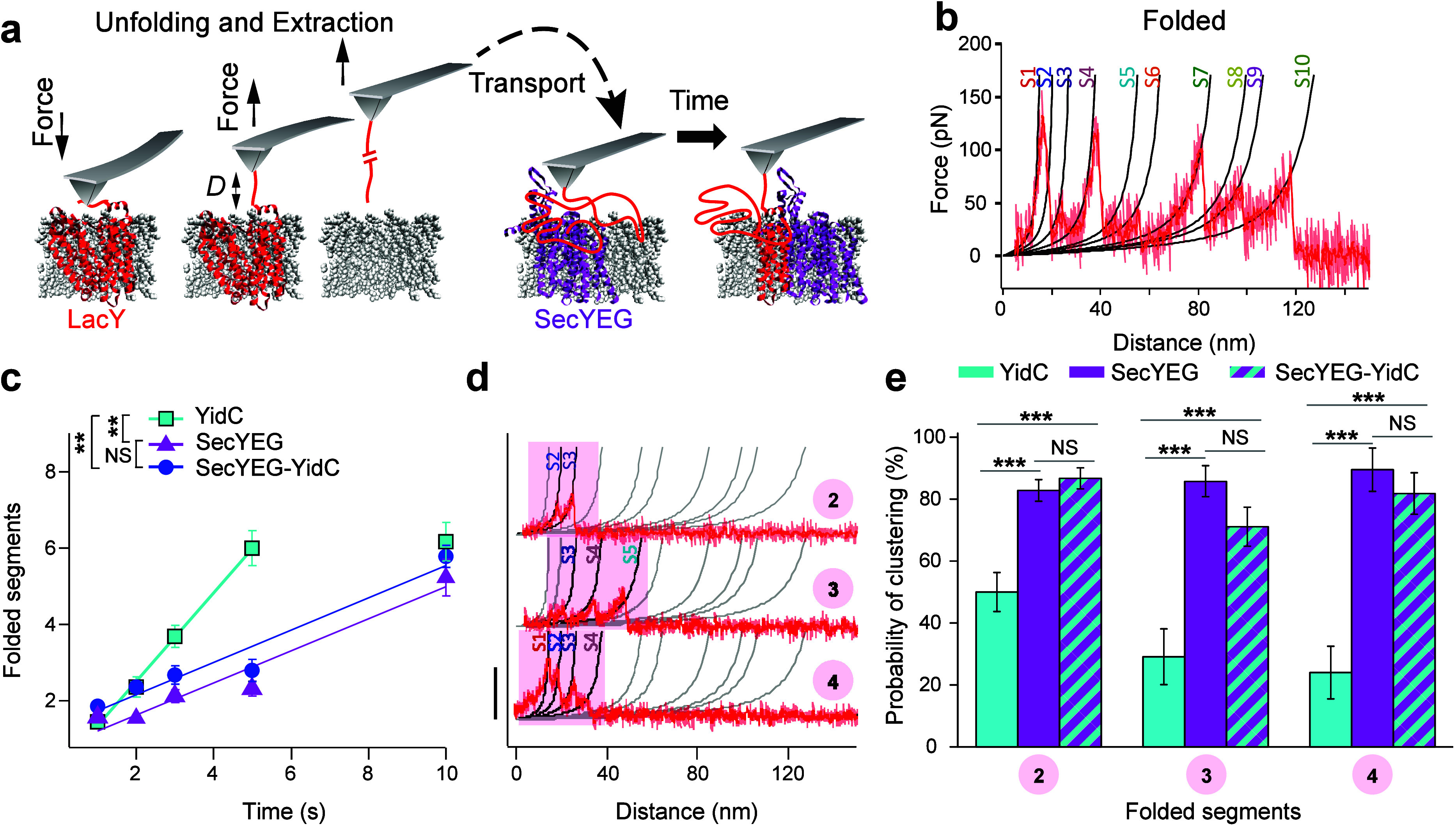
YidC- and SecYEG-mediated insertion differ kinetically and mechanistically. (a) Schematic of the insertion and folding experiment of a completely unfolded LacY polypeptide into a target membrane containing SecYEG. An AFM stylus unspecifically attaches the C terminus, mechanically unfolds and extracts LacY from the membrane, and then holds the unfolded polypeptide ∼5–10 nm above a SecYEG-containing phospholipid membrane for defined times. (b) Force–distance curve recorded in the presence of SecYEG after 10 s, showing the complete insertion and refolding of LacY. (c) Folding kinetics of LacY mechanically unfolded in the presence of YidC, SecYEG, or a SecYEG–YidC fusion complex. Colored linear fits represent insertion and folding rates of structural segments. (d) Force–distance curves showing single LacY polypeptides inserting and folding 2, 3, or 4 adjacent structural segments in the presence of SecYEG. WLC fits matching individual force peaks are shown in black, while the other WLC fits taken from the native LacY unfolding fingerprint are shown in gray. (e) Frequencies of detecting the insertion and folding of 2, 3, or 4 structural segments in the presence of of YidC, SecYEG, or a SecYEG-YidC fusion complex. Data were obtained from 478 (YidC), 395 (SecYEG), 397 (SecYEG-YidC fusion) force–distance curves. Statistical significance was assessed using ANCOVA (c) and Z-tests (e): *P* > 0.05 (NS), *P* < 0.05 (*), *P* < 0.01 (**), *P* < 0.001 (***). Error bars show S.E.. Adapted with permission from ref.^57^ Copyright 2019 The American Association for the Advancement of Science.

Moreover, unlike YidC, which inserts stable structural segments in a stochastic order, SecYEG supports the insertion of structural segments in a sequential manner after a first segment has been inserted (Figure 10d,e, Figure 11). These findings highlight fundamental differences in how the SecYEG translocon and the YidC insertase insert and fold membrane proteins into lipid bilayers.

**Figure 11:**
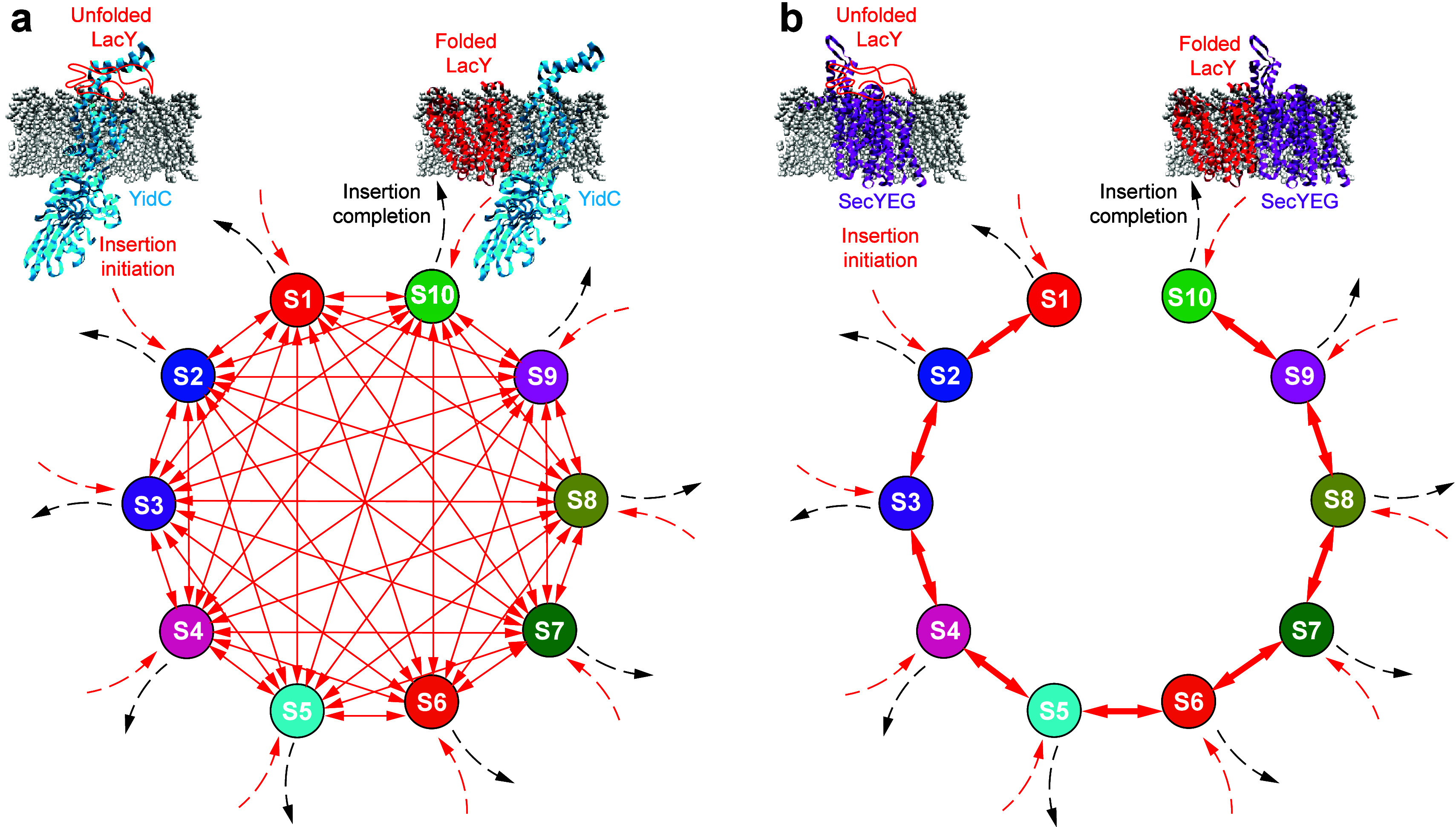
SecYEG and YidC assist the insertion and folding of LacY into a phospholipid membrane via distinct pathways. (a) YidC initiates LacY insertion and folding from any structural segment, after which the remaining segments (S1–S10) are then randomly inserted until folding is completed. (b) SecYEG, alone or together with YidC, also starts insertion and folding from any segment but proceeds sequentially. Dashed red arrows mark possible starting points of insertion, red double arrows indicate insertion and folding steps, and dashed black arrows show completion. Reproduced with permission from ref.^57^ Copyright 2019 The American Association for the Advancement of Science.

Biochemical studies indicate that in cells LacY is inserted cotranslationally into the cytoplasmic membrane through the signal recognition particle (SRP)-dependent SecYEG translocon. YidC interacts with LacY during biogenesis, but it primarily promotes proper folding of the transmembrane domains, acting as a membrane-embedded chaperone.^52,55^ Depletion of YidC perturbs LacY folding, demonstrating the cooperative roles of SecYEG and YidC in membrane protein biogenesis. Consistent with this view, SMFS experiments in reconstituted systems have shown that YidC protects LacY from misfolding and can even mediate its insertion independently under *in vitro* conditions, although when both, YidC and SecYEG, are present SecYEG generally dominates the insertion process.^57^ To directly examine this process, a SecYEG–YidC fusion construct was engineered and reconstituted into the acceptor membrane for AFM-based pull-and-paste assays of single membrane proteins. The SecYEG–YidC fusion construct inserted and folded LacY similarly to SecYEG alone (Figure 9), thus indicating that the insertion is driven primarily by SecYEG.^57^ As observed for SecYEG alone, the sequence of force peaks recorded upon unfolding of the folded LacY substrate suggested that structural segments insert and fold sequentially. These findings are consistent with a higher functional affinity of LacY for SecYEG, supporting the view that the SecYEG translocon is the principal mediator for the insertion of multispanning membrane protein into the inner membrane.^139,140^ In this context, YidC likely serves a supportive role like a chaperone by preventing misfolding and stabilizing nascent structural segments that would otherwise destabilize and misfold.

It is interesting to note that the pull-and-paste assay can, in principle, be broadly adapted to study other molecular players that assist membrane protein insertion and folding. For instance, soluble chaperones or small molecules can be incorporated into a pull-and-paste assay. This approach could also be extended to mimic the membrane protein insertion assisted by the ribosome. For example, having the C-terminus of membrane proteins attached to the AFM tip and their N-terminus being free to interact with the membrane mimics a physiological scenario where membrane protein precursors are translated starting from the N-terminus. Beyond fundamental studies, the pull-and-paste assay can be applied to synthetic biology applications. Membrane proteins could in principle be mechanically reconstituted into membranes of interest at high spatial precision within the membrane, allowing control over topology, density, and combinations of different proteins.

## 6 Assisted Insertion and Folding of β-Barrel Membrane Proteins

### 6.1 General Architecture of β-Barrel Membrane Proteins

Transmembrane β-barrel proteins are structurally and mechanistically distinct from their α-helical counterpart. Unlike the latter, which traverse the lipid bilayer through one or more membrane-spanning α-helices, transmembrane β-barrel proteins span the lipid bilayer by a series of antiparallel β-strands.^141,142^ The β-strands form an extended β-sheet that rolls into a cylindrical β-barrel structure surrounding a central cavity. This architecture is unique to outer membranes and is found almost exclusively in Gram-negative bacteria, as well as in the outer membranes of mitochondria and chloroplasts, reflecting their evolutionary origin from endosymbiotic Gram-negative bacteria.^143^ The β-barrel architecture is not only a structural solution to the demands of the outer membrane but a direct consequence of their biogenetic pathways.^144,145^ Unlike α-helical transmembrane proteins in bacterial inner membranes, β-barrel outer membrane proteins follow a more complex biogenetic route.^76^ After synthesis in the cytoplasm, outer membrane proteins are first translocated across the inner membrane by the Sec translocon and subsequently traverse the periplasmic space before insertion into the outer membrane. Critically, due to its dual role as an insertase of inner as well as a translocase of outer membrane proteins the Sec translocon cannot efficiently translocate sequences that resemble hydrophobic α-helical transmembrane domains but rather inserts these laterally into the inner membrane.^146^ Thus, in order to avoid insertion into the inner membrane, outer membrane proteins must not contain long, contiguous hydrophobic α-helices. While an α-helix typically requires ≈20–25 largely hydrophobic amino acids to span the ≈5 nm thick lipid membrane, an extended β-strand only needs 8–12 residues for the same distance.^147^ Moreover, with only every other residue facing outward from the β-barrel, the overall hydrophobicity of these segments can be comparatively low, allowing them to evade recognition by the Sec machinery as potential transmembrane segments.^137^

Despite their reliance on a simple repeating structural building block, β-barrel membrane proteins display remarkable structural and functional diversity. Most β-barrels are composed of an even number of β-strands, ranging from small 8-stranded β-barrels such as OmpA, OmpX, and PagP, to large β-barrels like the 26-stranded LptD,^148^ and the 36-β-stranded SprA,^149^ the largest known β-barrel to date in Gram-negative outer membranes. While far less common, also uneven numbers of β-strands exist, such as in the 19-β-stranded voltage-dependent anion channel (VDAC) in mitochondria.^150^ Most β-barrel outer membrane proteins adopt a topology in which both the N- and C-termini face the periplasmic space. Functionally, β-barrel membrane proteins participate in a broad array of processes including passive diffusion, substrate-specific and mechanically gated channel activity, enzymatic catalysis, protein translocation, and membrane anchoring. Many also serve as key structural components of secretion systems, fimbriae, and pili, or form covalent contacts with the peptidoglycan cell wall.

### 6.2 The Gram-Negative β-Barrel Assembly Machinery (BAM) Complex

In Gram-negative bacteria the folding and insertion of β-barrel outer membrane proteins is mediated by the β-barrel assembly machinery (BAM) complex, a conserved multiprotein system centered around the essential insertase BamA.^66^ BamA consists of a 16-β-stranded transmembrane β-barrel connected to five periplasmic polypeptide-transport associated (POTRA) domains that coordinate interactions with incoming substrate proteins and other BAM components. Accessory lipoproteins BamB, BamC, BamD, and BamE associate with BamA on the periplasmic side, facilitating substrate recognition and transfer.^70,151^ While the precise mechanism by which the BAM complex catalyzes membrane insertion remains an area of ongoing investigation, current models propose BamA to transiently open its lateral gate formed by the first and last β-strands of the β-barrel to receive incoming β-strands from client proteins.^152^ This process likely involves the formation of a hybrid β-barrel intermediate, where incoming client β-strands augment the BamA β-barrel, followed by stepwise closure and release into the outer membrane.^153^ Curiously, the biogenesis of β-barrel proteins in mitochondria and chloroplasts involves an additional translocation step. Following cytosolic synthesis by the host cell, these proteins are first translocated across the mitochondrial outer membrane prior to insertion into the outer membrane from the inter membrane space, a seemingly circuitous pathway dictated by the location and specificity of the evolutionarily conserved Omp85-family insertases Sam50 in mitochondria, and Toc75 in chloroplasts. Although the precise mechanism of insertion by β-barrel insertases remains debated,^154–157^ the general folding pathways of transmembrane β-barrel proteins have been biophysically characterized in great detail.

### 6.3 Mechanical Unfolding of β-Barrel Membrane Proteins

Over the recent decades the mechanical unfolding and folding of several transmembrane β-barrel proteins have been probed by SMFS. Exposed to mechanical force, transmembrane β-barrel proteins across scales show a highly reproducible unfolding behavior, where individual unfolding steps are almost exclusively determined by β-hairpins. The outer membrane protein G (OmpG), a 14-β-stranded monomeric β-barrel outer membrane protein of *E. coli*, displays an unfolding fingerprint consisting of 7 distinct force peaks, each corresponding to the unfolding of one β-hairpin.^115^ Similarly, the 18-stranded β-barrel of the trimeric *E. coli* maltose uptake channel maltoporin (LamB) unfolds via 9 distinct unfolding steps shaped by individual β-hairpins,^158^ as does the 19-β-stranded voltage-dependent anion channel (VDAC) from mitochondria, with the only exception of β-hairpins 1 and 2 unfolding in a single step.^159^ Even the large 22-β-stranded β-barrel of the *E. coli* ferric hydroxamate uptake receptor (FhuA) unfolds via 11 steps determined by its 11 β-hairpins.^160^

One exception is the small 8-stranded β-barrel of the outer membrane protein A (OmpA) from *Klebsiella pneumoniae*, which unfolds first via a set of 3 β-strands, then unfolds sequentially two β-hairpins, and finally a single β-strand.^161^ Curiously, a similar alteration switching the unfolding steps from two β-hairpins to a set of three as well as a single β-strand has been observed in the unfolding pathway of OmpG in a pH-dependent manner.^116,127^ Nevertheless, the archetypical unfolding feature of β-barrel proteins is the β-hairpin, which is mainly owed to two reasons: Primarily, the sequential unfolding behavior via β-hairpins is well-suited to the sequential architecture of the β-barrel. Second, the constriction of the protein by the surrounding membrane, which aligns the protein relative to the mechanical unfolding direction applied through SMFS, dictates this unfolding sequence.^102^ The latter is explained by the mechanical force-induced unfolding, which determines which unfolding pathway a protein takes across a rugged energy landscape, as highlighted by MD simulations comparing the mechanical unfolding of OmpG embedded in a membrane with the mechanical unfolding of the water-soluble β-barrel of green fluorescent protein (GFP).^162^ Thereby, the unfolding steps of a β-barrel protein curiously disappear once the constriction of the membrane has been removed, allowing the protein to spatially reorient to offer the least possible resistance against the unfolding force and thus follow a smooth path across the free-energy landscape.^162^ This unique unfolding behavior may be further supported by the aqueous environment, which allows rapid hydration of newly exposed protein surfaces and weakens hydrogen-bond networks that are otherwise stabilized in the weakly competitive hydrogen-bonding environment of the membrane bilayer.^8^

### 6.4 Mechanical (Re)­folding of β-Barrel Membrane Proteins

The reproducibility found in the mechanically induced unfolding patterns of β-barrels, namely the sequential unfolding via β-hairpins, lends itself to an excellent platform to characterize their refolding.^102^ Thereby, much as previously described for α-helical membrane proteins, several β-barrel membrane proteins have been shown to possess the ability to reinsert β-strands into the lipid bilayer. This ability has first been described for the 12-stranded β-barrel of OmpG, which after partial unfolding and subsequently relaxation in close proximity to the membrane, can fully refold into the membrane within a time frame of 5 s.^163^ Thereby, much like the unfolding steps, the folding steps are shaped by individual β-hairpins. However, the order in which these β-hairpins insert and fold into the bilayer is not the reverse order of unfolding. Instead, the main folding pathway appears to be initiated at β-hairpin IV, followed by β-hairpins V and VI, before folding of the remaining β-hairpins III, II, and I.^163^ The stepwise folding pathway of OmpG somewhat contrasts folding mechanics of the smaller 8-β-stranded β-barrel of OmpA. Much like OmpG, OmpA possess the ability to insert and fold into the lipid bilayer to readopt a native-like fold, albeit on slightly shorter time scales, with only about 2 s required for the β-barrel to form.^161^ Yet, a stepwise folding pathway as in the case of OmpG was not observed for OmpA, which instead appeared to comply with a two-state model of folding, characterized by a concerted insertion and folding mechanism of the previously entirely unfolded polypeptide.^164,165^ Yet, this interpretation might be owed to fast folding kinetics masking a stepwise insertion and folding mechanism due to limitations in the time-resolution of the SMFS-based refolding experiment. The mechanism driving the folding of transmembrane β-barrel proteins gets further obscured by SMFS experiments probing the refolding of the large 22-β-stranded β-barrel of FhuA.^78,160^ Unlike the smaller OmpA and OmpG, the large β-barrel of FhuA appears to lack the ability to insert and fold into the membrane in a self-guided process. Instead, when mechanically partially unfolded from the lipid membrane and subsequently relaxed in close proximity to the membrane the FhuA polypeptide adopts a range of misfolded conformations, as evidenced by the appearance of novel unfolding steps which bear no similarity to the (un)­folding intermediates observed upon unfolding the native protein.^160^ Taken together, the mechanical folding experiments with β-barrel outer membrane proteins of different sizes suggest that upon a certain level of complexity β-barrel insertion and folding can no longer occur in a self-guided process.

The lack of refolding in FhuA makes this protein an excellent client to characterize the effects of additional folding factors on the folding process (Figure 12). In *E. coli* within a vast periplasmic protein quality control network two specific chaperones, the trimeric 17 kDa protein (Skp) and the survival factor A (SurA), have been implied to be responsible for facilitating the passage of β-barrel outer membrane proteins across the aqueous periplasm. Thereby, SurA serves as the main folding factor for outer membrane proteins, while Skp potentially acts as a backstop rescuing proteins fallen off the main folding pathway.^77^ Indeed, when mechanically unfolded and afterward refolded in the presence of either Skp or SurA the occurrence of misfolded conformations of FhuA greatly reduces.^78^ Yet, the two chaperones impact membrane insertion and folding of FhuA differently, in line with their intended roles along the cellular folding pathway.^81^ Whereas the presence of Skp suppresses the formation of any folded state and instead stabilizes an unfolded conformation, the presence of SurA promotes the insertion and folding of correctly folded intermediates.^78^ Thereby folding occurs via the sequential folding of individual β-hairpins in agreement with the self-guided stepwise folding pathway previously described for OmpG.^163^ Kinetically, membrane insertion and folding of FhuA in the presence of SurA is initiated within the first second following relaxation of the unfolded FhuA polypeptide and requires a minimum of 10 s to fold the complete β-barrel.^78^ While the order in which β-hairpins fold is not strictly defined, the probability for β-hairpins to fold increases the closer they locate to the membrane anchor.

**Figure 12:**
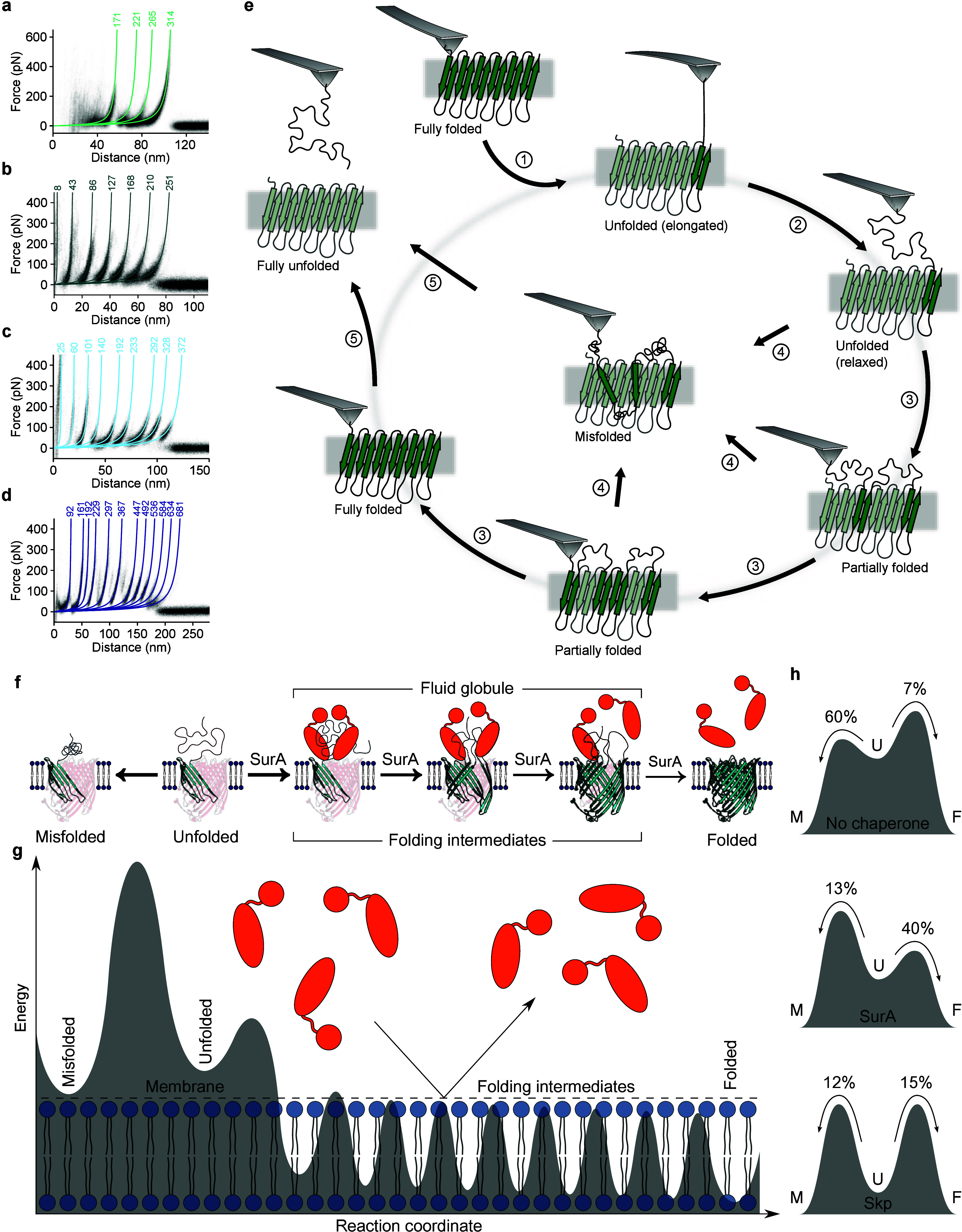
Unfolding and refolding of β-barrel membrane proteins. (a–d) Density plots of multiple superimposed force–distance curves recorded during mechanical unfolding of single (a) OmpA, (b) OmpG, (c) LamB, and (d) FhuA proteins from lipid membranes using AFM-based SMFS. WLC model curves of the contour lengths of the force peaks (numbers of amino acids) indicate the length of the structural segments being unfolded. (e) The insertion and folding pathway of β-barrel membrane proteins observed by single-molecule force spectroscopy. A β-barrel membrane protein is partially unfolded by applying a mechanical force to one terminus (1). The unfolded polypeptide is then relaxed (2). From the relaxed state, the unfolded polypeptide can refold in the lipid bilayer in a series of steps, which are shaped by the sequential insertion of individual β-hairpins (3). At any point along the folding pathway, complex β-barrel proteins can diverge from the main folding pathway and adopt a misfolded conformation. The final unfolding (5) determines whether the β-barrel membrane protein had attained a folded state or was trapped in a misfolded conformation (4). (f–h) Folding pathways and free-energy landscape of FhuA receptors. (f) Insertion and folding pathways of FhuA in the absence of chaperones and in the presence of SurA (orange). Without chaperones, the majority of unfolded FhuA receptors misfold. SurA, when present, stabilizes the unfolded state of FhuA and promotes stepwise insertion and folding of β-hairpins in the lipid membrane. (g) Hypothetical folding free-energy landscape of FhuA in the presence of SurA. SurA (orange) is spatially excluded from the lipid membrane (blue). Each β-hairpin inserted into the lipid membrane is stabilized by a free-energy well. The lipid membrane thus acts as a free energy sink for the insertion of β-hairpins and physically separates transient folds from the chaperones. (h) Modulation of the folding free-energy landscape by chaperones. The free-energy barriers separating the unfolded (U) from the misfolded (M) and folded (F) states, as approximated from folding probabilities of single FhuA refolding experiments conducted at 1 s refolding time. Figure panels a–e reproduced with permission from ref.^102^ Copyright 2018 Annual Reviews. Figure panels f–h reproduced with permission from ref.^78^ Copyright 2015 Springer Nature.

In summary, mechanical refolding experiments on the bacterial transmembrane proteins OmpA, OmpG, and FhuA suggest that their folding and membrane insertion can proceed from a relaxed, unfolded conformation into a fully folded β-barrel along a self-guided stepwise folding pathway shaped by the insertion of individual β-hairpins. This self-guided refolding process is effective for small to moderately sized β-barrels and is facilitated by the energetic landscape provided by the hydrophobic membrane environment. However, as protein size and structural complexity increase, as exemplified by FhuA, spontaneous refolding becomes inefficient or fails entirely. In such cases, the prevention of misfolded conformations by external folding factors becomes essential for successful membrane integration. The observation that small β-barrels like OmpA and OmpG can fold without assistance, whereas larger ones like FhuA require additional folding factors, points to a threshold of structural complexity beyond which autonomous folding fails. However, the determinants of this threshold are not yet understood. It remains unclear whether the inability to self-insert and -fold into the membrane is governed by structural factors such as the number of β-strands, β-strand topology, β-barrel curvature, or loop complexity, or whether there are critical folding intermediates in larger OMPs that require stabilization by chaperones. In this context, previous SMFS experiments highlight the importance of the hydrophobic membrane environment itself, which can act as a free-energy sink to stabilize and spatially separate membrane-inserted folding intermediates (namely β-hairpins) from the surrounding aqueous environment. Bulk studies suggest that the lipid environment, including bilayer thickness, curvature, charge, and fluidity, plays a crucial role in facilitating the insertion and folding of β-barrel proteins. Yet, it remains largely unexplored how specific lipid properties shape folding pathways at the single-molecule level, whether certain lipids stabilize intermediate β-hairpin states, and how membrane asymmetry affects folding energetics. While SMFS has revealed that some β-barrel membrane proteins can fold independently, the majority of outer membrane proteins *in vivo* rely on the β-barrel assembly machinery (BAM) complex. It remains to be determined how the BAM complex shapes the folding pathways, energetics, and intermediate states of transmembrane β-barrels. A key technical challenge will be the development of SMFS-compatible systems that provide experimental access to functional BAM complexes in planar lipid membranes, which would allow the direct comparison of BAM-assisted and spontaneous folding for outer membrane proteins, as well as the identification of insertion and folding intermediates stabilized (or destabilized) by BAM components.

## 7 OMVs as a Native Platform to Study Membrane Protein Folding

In Gram-negative bacteria the outer membrane is surrounded by a protective layer, which forms a highly specialized environment that is both chemically and mechanically distinct from most other biological membranes.^166^ Unlike most cellular membranes, including the plasma membranes of bacteria as well as of eukaryotic cells, which are predominantly composed of phospholipids, outer membranes contain a high fraction of lipopolysaccharides (LPS) in a highly asymmetric arrangement.^167^ Whereas the inner leaflets of outer membranes are composed of phospholipids in ratios similar to those of bacterial inner membranes, their outer leaflets are composed exclusively of LPS. This extreme lipid asymmetry makes it extremely difficult to reconstitute membrane proteins purified from bacterial outer membranes into lipid bilayers that would be needed to characterize their thermodynamic stability and folding pathways in a native-like membrane environment. Thus, far, all SMFS experiments investigating β-barrel unfolding and folding relied on their reconstitution into liposomes composed of synthetic lipids or lipid extracts. These systems, while useful, lack the asymmetric and LPS-rich architecture of native outer membranes. Recently, outer membrane vesicles (OMVs) released by Gram-negative bacteria have emerged as a native platform to overcome this limitation.^108^ OMVs offer a noninvasive method to isolate bacterial outer membranes in their native asymmetry. During growth bacteria shed fragments of their outer membranes in the form of OMVs, which have the same membrane composition as the parental outer membrane and are filled with periplasmic components.^168^ OMVs can be readily isolated from bacterial culture supernatants, providing access to intact, asymmetric outer membranes without the need for reconstitution.^169,170^ By overexpressing outer membrane proteins in specific *E. coli* strains such as porin-depleted BL21­(DE3)­omp8 the proteome of the OMVs produced by the bacteria can be produced toward highly enrichment of selected proteins.^108^ Thereby, the use of porin-deficient strains can reduce endogenous major porins, thus enabling the predominant characterization of specific target proteins. Thus, OMVs were first used to characterize the mechanical unfolding of β-barrel membrane proteins directly from their physiological membrane.^108^ As typically both the N- and C-termini of these proteins face the periplasm, they are accessible for attachment to the AFM tip while maintaining their native topology, making OMVs an ideal system for single-molecule force spectroscopy experiments. Furthermore, OMVs allowed, for the first time, detailed mechanical investigations of BamA in a native membrane context.

The initial characterization of the mechanical unfolding behavior of the transmembrane β-barrel proteins OmpG, FhuA, Tsx, and BamA from the native membranes of OMVs revealed that the predominant unfolding pathway, proceeding via sequential extraction of individual β-hairpins, was preserved across synthetic and native membrane environments (Figure 12).^108^ Both OmpG and FhuA exhibited largely identical unfolding behaviors when unfolded from native membranes or proteoliposomes made from *E. coli* polar lipid extract. Similar experiments with Tsx and BamA reinforced the conclusion that OMVs preserve native folding, where Tsx unfolded via six unfolding steps corresponding to its six β-hairpins, and BamA showed a reproducible seven-step unfolding pattern, with β-hairpins 7 and 8 unfolding together in a single unfolding step.^108^ This consistent β-hairpin-wise unfolding pattern suggests that the mechanical and structural stability of these proteins are largely intrinsic and robust against variations in lipid composition. However, subtle differences emerged when comparing the unfolding behavior of FhuA across native and reconstituted membranes, where an additional force peak appeared in OMV-derived force–distance curves, which was absent in reconstituted samples. This defined an alternative unfolding pathway that coincided with β-hairpins 4 and 5 in a region that has been described to contain an LPS-binding site,^171^ suggesting that interactions with LPS in native membranes can modulate the free-energy landscape of β-barrel proteins. While much remains unknown about how the complex, asymmetric lipid environment of the bacterial outer membrane shapes the folding and stability of β-barrel proteins, recent work has begun to uncover how key components of the membrane protein biogenesis machinery behaves mechanically in this native context.^172,173^ In particular, two subsequent studies provided detailed insights into the mechanical stability of the outer membrane insertase BamA in response to both intrinsic features such as domain organization and extrinsic factors including membrane composition.^172,173^

In the first study, full-length BamA as well as truncated and mutant variants were mechanically unfolded to analyze the contribution of its POTRA domains, its extracellular lid structure, and the native membrane composition of OMVs to β-barrel stability (Figure 13a–c).^172^ While it was found that BamA shows the typical stepwise unfolding behavior via β-hairpins, it was also observed that these unfolding pathways are highly sensitive to perturbations in the surrounding environment. Compared to other β-barrel membrane proteins the forces required to mechanically unfold BamA are relatively low. However, compared to BAM reconstituted in membrane bilayers made from *E. coli* polar lipid extract these unfolding forces further reduced by 15–25% when in the native membrane, in particular for β-hairpins 1 to 4.^172^ Importantly, such a reduction in membrane protein stability imposed by the native membrane environment had not been observed in previous experiments with OmpG and FhuA,^108^ suggesting that the lower mechanical stability of BamA in native membranes may have functional implications. However, mechanical unfolding of a truncated BamA variant lacking all five POTRA domains resulted in a similar reduction in unfolding forces, suggesting that the POTRA domains contributed to stabilize the proximal β-hairpins, as did a single point mutation in the conserved lid-lock residue R661 in extracellular loop 6.^172^ Taken together, the data illuminated how both local and global structural elements influence the mechanical stability of BamA, supporting a model of BamA as a metastable β-barrel,^156^ whose conformational flexibility is potentially essential for its role in outer membrane protein biogenesis.

**Figure 13:**
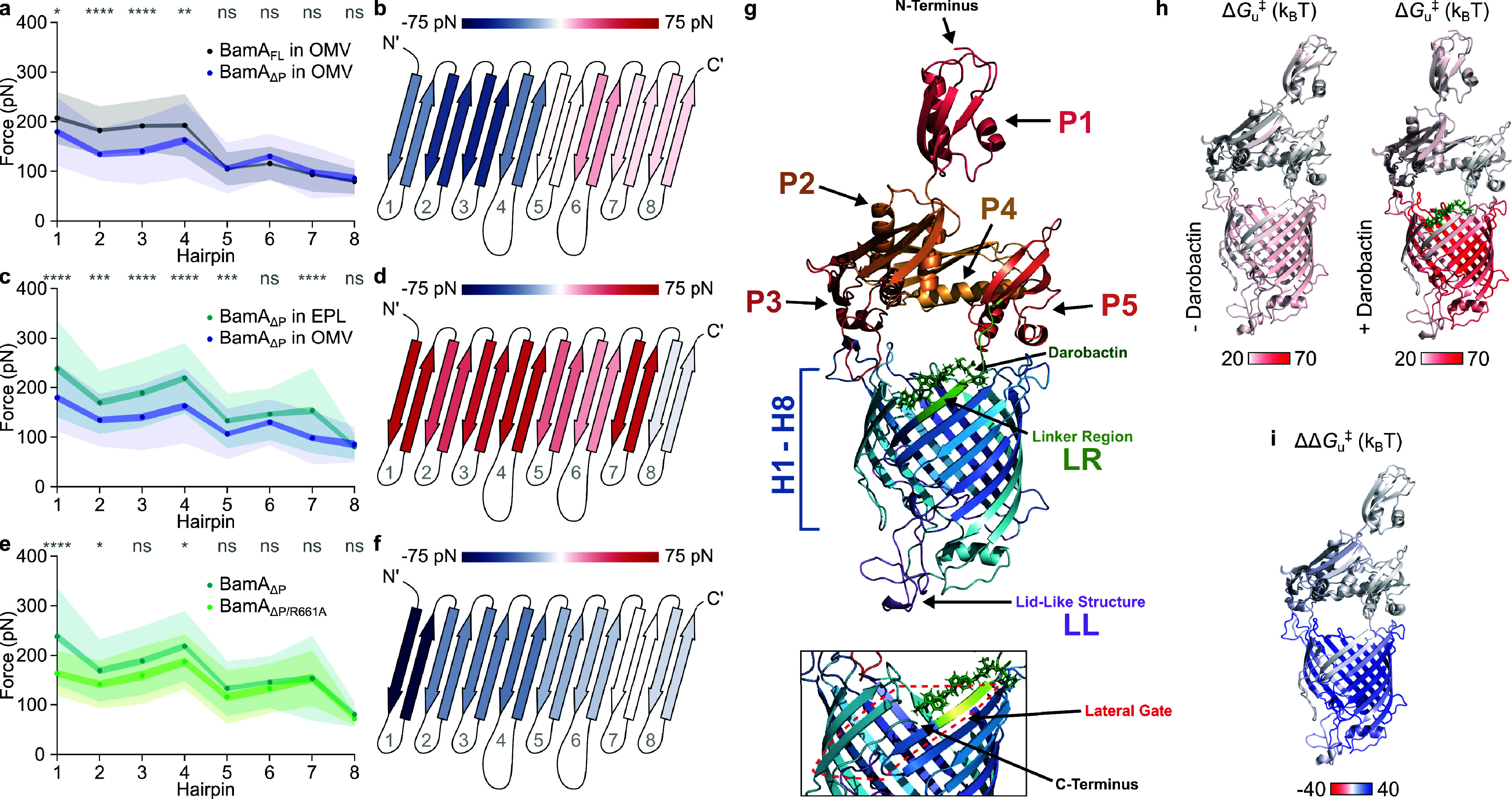
Modulation of the mechanical stability of the BamA β-barrel embedded in the native lipid environment of the outer membrane vesicles (OMVs). (a, b) Mechanical stability changes of BamA caused by removal of the POTRA domains. Mean unfolding forces (circles) of β-hairpins 1–8 of full-length BamA and BamAΔP, mapped to the secondary structure cartoon of BamA. The color of β-hairpins represents the difference in force required to mechanically unfold BamAΔP compared with full-length BamA. (c, d) Mechanical stability changes caused by changing the membrane environment. Mean unfolding forces (circles) of β-hairpins 1–8 of BamAΔP from reconstituted membranes made from *E. coli* polar lipid extract and native membrane of OMVs, mapped to the secondary structure cartoon of BamA. The color of β-hairpins represents the difference in force required to mechanically unfold BamAΔP from membranes made from *E. coli* polar lipid extract compared with native membrane of OMVs. (e, f) Mechanical stability changes caused by the lid-lock of BamA. Mean unfolding forces (circles) of β-hairpins 1–8 of BamAΔP and BamAΔP/R661A, mapped to the secondary structure cartoon of BamA. (g–i) Effect of darobactin on the mechanical and energetic properties of BamA. (g) Structure of BamA bound to darobactin (PDB: 7NRI^119^). Darobactin is shown in green. Colored segments of BamA represent force peak classes, which were assigned to structural segments. Both termini are indicated in black. Inset at the bottom shows the lateral gate of BamA, indicated by the red area. (h) Gibbs free energy difference between the folded and the transition state along the unfolding intermediates described by the unfolding pathways (free-energy landscape) of unliganded (PDB: 5D0O^93^) and to the darobactin-bound BamA structure. (i) Darobactin-induced differences in the free-energy landscape relative to the unliganded state mapped to the BamA structure. Figure panels a–f adapted with permission from ref.^172^ Copyright 2018 Elsevier. Figure panels g–i adapted from ref.^173^ under Creative Commons CC BY 4.0, Copyright 2022 Elsevier.

The second study that further advanced the mechanical understanding of BamA in the native membrane environment of OMVs explored how the natural antibiotic Darobactin modulates the conformational landscape of BamA at the single-molecule level using dynamic force spectroscopy (Figure 13g–i).^173^ Darobactin, a heptapeptide found in *Photorhabdus*, mimics the C-terminal beta-signal of BamA clients and seals the lateral gate of the BamA β-barrel formed by the first and last β-strand.^174,175^ In absence of Darobactin, unfolding of the β-barrel highlights that β-hairpins 1 to 4 are mechanically more stable than the following β-hairpins 5 to 8, reflecting a gradient in stability and flexibility aligned with the lateral gate architecture of BamA (Figure 13h). Upon introduction of Darobactin, the general β-hairpin wise unfolding pattern remained; however, unfolding forces of the β-barrel region increased significantly, most prominently for β-hairpins 1 to 4, whereas the POTRA domains remained largely unaffected.^173^ Therefore, Darobactin binding at the lateral gate of BamA induces an allosteric mechanical stabilization across the β-barrel, substantially reshaping its free-energy landscape and enhancing the energetic and kinetic stability of the protein’s transmembrane region. These changes effectively lock BamA in a mechanically rigidified state, effectively eliminating the functional dynamics required for client insertion. Strikingly, the degree and magnitude of mechanical stabilization induced by Darobactin binding closely mirrors the stabilization observed when comparing BamA unfolded from the native OMV membrane to that from bilayers composed of *E. coli* polar lipid extract.^172^ Although a direct mechanistic link has yet to be demonstrated, this parallel raises the possibility that reconstituted systems, which lack key features such as lipid asymmetry and LPS content, may inadvertently trap BamA in a nonphysiological, overly stabilized conformation. Such stabilization, while beneficial for biophysical measurements, could therefore obscure the structural flexibility required to gain a detailed understanding of BAM-mediated outer membrane protein insertion. This underscores the importance of preserving the native membrane context when probing the folding, dynamics, and activity of β-barrel assembly factors.

## 8 Conclusions and Outlook

AFM-based SMFS provides a powerful approach to the detailed folding and insertion mechanisms and pathways of membrane proteins under near-physiological conditions. Precisely mapping the mechanical folding pathways of membrane proteins with SMFS allows the detection of unfolding and folding events at the resolution of single secondary structural segments and enables the reconstruction of the unfolding and folding free-energy landscapes, which are inaccessible to ensemble techniques. Thereby, SMFS has begun to reveal recurring principles governing the folding of membrane proteins and highlight the mechanistic diversity of the cellular machineries acting along the folding pathways of membrane proteins.

In α-helical membrane proteins, exemplified by LacY, the lipid environment emerged as a decisive determinant of structural stability and transmembrane topology, with specific lipid compositions promoting either native or non-native folds. YidC was shown to act not only as an insertase but also as a holdase chaperone, reducing misfolding events and promoting the stochastic, stepwise membrane insertion and folding of LacY. The function of YidC is contrasted by the SecYEG translocon, which directs LacY folding in a more sequential order, compared to the flexible insertion pathways promoted by YidC. For β-barrel membrane proteins, SMFS revealed a spectrum of unfolding and folding behaviors dependent on their structural complexity. Smaller transmembrane β-barrels such as OmpA and OmpG after being partially unfolded can refold autonomously through the self-guided, stepwise insertion of β-hairpins, whereas larger β-barrels like FhuA fail to fold spontaneously and require the assistance of chaperones such as SurA or Skp. These observations point to a structural complexity threshold beyond which autonomous membrane insertion and folding becomes unfeasible. The latter further necessitates specialized machineries like the outer membrane BAM complex. In this context the introduction of OMVs as an experimental platform provides a critical step forward, allowing β-barrel membrane protein insertion and folding and BamA function to be probed in the native asymmetric, LPS-rich environment of bacterial outer membranes.

Taken together, AFM-based SMFS stands out as a unique method for connecting the molecular details of membrane protein folding pathways with a physiological context. One principle emerging from the discussed examples is that membrane protein folding is not an isolated process but the result of a dynamic interplay between intrinsic features of the protein, the lipid environment, and specialized cellular machineries. Whereas simple topologies may exploit the membrane itself as an energy funnel for folding,^163^ an increasing topological complexity necessitates the targeted assistance of insertases, translocases, or chaperones to prevent misfolding and ensure correct integration into the membrane.^57,78^ AFM-based SMFS has proven instrumental in revealing this interplay by enabling the physical manipulation of membrane proteins in and outside of near-native membrane systems while closely mimicking physiological conditions, such as temperature and buffer solution.

Despite these advances, several fundamental questions underpinning the insertion and folding processes of membrane proteins remain unresolved. The precise free-energy barriers and rate-limiting steps that govern the membrane insertion and folding of different classes of membrane proteins remain largely ambiguous. Moreover, how native membrane features from bilayer asymmetry, curvature, and local thinning to membrane tension and lipid phase separation reshape insertion energetics is only beginning to be understood. AFM-based SMFS is uniquely positioned to address these challenges in the future and decipher the energetic principles underlying the fate of membrane proteins along their complex folding pathways. In particular, the role of mechanical factors impacting membrane protein folding—nascent chains are thought to fold under mechanical tension in the cotranslational folding pathway^86,176^ —are prone to be mimicked by SMFS. While the characterization of cotranslational folding has been achieved for water-soluble globular proteins, demonstrating vectorial folding and mechanical tension along the nascent chain,^177,178^ extending such approaches to membrane proteins may require the development of suitable (potentially planar) membrane systems that are compatible with ribosome-associated folding measurements to probe how nascent chains interact with the lipid bilayer during synthesis.

Nevertheless, the range of client membrane proteins, whose mechanical folding behavior has been characterized in SMFS-based experiments, remains quite limited. It thus remains to be determined to what extent the observations and conclusions drawn from AFM experiments utilizing mostly bacterial model proteins can be extrapolated to a more general picture of membrane protein folding. Extending these experiments to a broader range of substrates and conditions will be needed to distinguish universal principles from system-dependent phenomena and differentiate the mechanistic strategies of insertases from client-specific folding behavior. Thereby, complementarity with a broad range of biophysical methods such as cryo-EM, NMR spectroscopy, and molecular dynamics simulations will be vital to integrating the unique insights on the mechanical barriers, stochasticity, and heterogeneity of membrane protein folding determined by SMFS in the broader cellular context of protein biogenesis.
